# Quasi-hydrostatic equation of state of silicon up to 1 megabar at ambient temperature

**DOI:** 10.1038/s41598-019-51931-1

**Published:** 2019-10-29

**Authors:** Simone Anzellini, Michael T. Wharmby, Francesca Miozzi, Annette Kleppe, Dominik Daisenberger, Heribert Wilhelm

**Affiliations:** 1 Diamond Light Source Ltd, Diamond House, Harwell Science and Innovation Campus, Didcot, OX11 0DE UK; 2 PETRA III, Deutsches Elektronen-Synchrotron (DESY), Notkestrabe 85, 22607 Hamburg, Germany; 3 Sorbonne Université, UMR CNRS 7590, Muséum National d‘Histoire Naturelle, Institut de Minéralogie, de Physique des Matériaux et de Cosmochimie, IMPMC, 75005 Paris, France; 4 Helmoltz Institut Ulm, Helmhotzstrβe11, D-89081 Ulm, Germany

**Keywords:** Materials science, Physics

## Abstract

The isothermal equation of state of silicon has been determined by synchrotron x-ray diffraction experiments up to 105.2 GPa at room temperature using diamond anvil cells. A He-pressure medium was used to minimize the effect of uniaxial stress on the sample volume and ruby, gold and tungsten pressure gauges were used. Seven different phases of silicon have been observed along the experimental conditions covered in the present study.

## Introduction

Silicon is one of the most studied elements in the world for its use in microelectronics, semiconductors technologies and its importance in Earth science^1,2^. For example, it is believed that a certain percentage of Si could be contained in the Earth’s core together with Fe and Ni and other light elements (such as S, O, H and C)^3^. For these reasons, Si has been extensively studied (experimentally and theoretically) under extreme conditions and so far up to seven different polymorphs have been observed in compression experiments at ambient temperature.

At ambient conditions Si presents a diamond structure ($Fd\overline{3}m$) labeled as Si-I. Under increasing pressure, Si-I transforms, via a coexistence region, into the metallic Si-II phase with a tetragonal *β*-Sn structure (*I*4_1_/*amd*)^4^. The observed transition pressure from Si-I to Si-II and the pressure range over which coexistence is observed, strongly depend on the experimental conditions and they are both affected by non-hydrostatic stresses. In Diamond Anvil Cell (DAC) experiments performed using different techniques (from X-ray diffraction (XRD) to Raman), the reported Si-I/II transition pressure varies from 8.8 to 12.5 GPa^5–10^. Under uniaxial compression, the reported phase transition has been shown to occur at 12 GPa along the [100] and at lower pressure along the [111] direction via electrical resistance measurement^11^. Under shock compression, instead, the transformation is observed in the 6–14 GPa pressure range^12–15^.

In a DAC experiment using a 4:1 methanol:ethanol mixture as pressure medium, McMahon *et al*. observed a new phase transition from the *β*-Sn structure to an orthorhombic phase (space group *Imma*) at a pressure of 14.4 GPa^6^. The new phase, named Si-XI, remains stable up to 16 GPa, when the structure transforms to a simple hexagonal (*P*6/*mmm*) phase named Si-V^6,10^. Under further compression, Si-V converts to the orthorhombic Si-VI phase (*Cmca*) at a pressure of 37.6 GPa. This phase was first observed by Olijnyk *et al*.^10^ and subsequently indexed as a large orthorhombic cell, containing 16 atoms, by Hanfland *et al*.^16^. In the pressure range 40–42 GPa, the structure undergoes a further phase transition to the Si-VII (*P*6_3_/*mmc*) form, which remains stable up to 79 GPa^17^. Above this pressure, the cubic phase Si-X ($Fm\overline{3}m$) develops which is stable up to at least 243 GPa^17^.

Although the characterization of the high temperature part of the phase diagram of Si is beyond the scope of the present paper, a comprehensive description of the experimental and theoretical works performed so far on Si can be found in the articles of Turneaure *et al*.^18^ and Paul *et al*.^19^. Under decompression at ambient temperature, four additional phases have been observed to form from Si-II. In particular, under rapid decompression of Si-II to atmospheric pressure, the tetragonal phases Si-VIII (*P*4_1_2_1_2) and Si-IX (*P*4_2_22) appear^20^. Upon relative slow decompression, Si-II transforms instead around 8–10 GPa into a semiconducting phase with a rhombohedral R8 structure (Si-XII)^21^ which in turn transforms after further decompression into the Si-III phase, with a body-centred cubic BC8 unit cell^22^.

Although Si has been extensively studied using several experimental and theoretical techniques, most of the studies performed so far were focused more on the determination of the pressure domain of a particular phase (or several phases)^5,16,23–25^. A synchrotron XRD characterization of the complete compression curve of Si at ambient temperature by modern DAC techniques has never been performed. To our knowledge, the most recent experimental determination of the Si equation of state (EOS) is from 1990^17^. In these experiments the Si EOS was investigated by energy dispersive XRD from 40 to 243 GPa without any pressure medium. Since then, great progress has been achieved in the accuracy of the determination of compression curves. In particular the use of helium as a pressure transmitting medium has meant that samples experience a higher hydrostatic stress during the experiment^26^. Third generation synchrotron sources have permitted smaller beam sizes with higher X-ray fluxes to be used, allowing for smaller sample sizes with more homogenous pressure in the illuminated region. Finally, the systematic uncertainties of DAC pressure metrology have been reduced, most importantly for the ruby pressure gauge^27^. In the last few years it has been shown that results obtained by modern synchrotron DAC techniques can significantly improve on those obtained by older methods^27–29^. These considerations have lead us to re-examine the Si compression curve at ambient temperature. To the best of our knowledge, this compression curve has never been investigated across the complete range from ambient to pressures greater than 1 MBar using the same method. By using helium (He) as a pressure transmitting medium we may observe changes in the sequence of phases formed, in line with those predicted by theory. Thus we will be able to better constrain the pressure domains of each phase under hydrostatic conditions.

## Results

The structural evolution of crystalline Si from ambient pressure to 105.2 GPa has been investigated at ambient temperature under quasi-hydrostatic conditions. Over this pressure range, Si was observed to undergo six phase transitions four of which are associated with a coexistence region. In the following sections, the stability field and compression curve of each phase are described and compared with previous studies. The crystallographic changes occurring in the structures and the corresponding mechanisms of the phase transitions are well known, therefore they are not discussed in this paper. A comprehensive description of the phase transitions of the group IV elements has been previously presented by Katzke *et al*.^30^.

### Si-I: diamond structure

At ambient pressure, the observed signal on the image plate presents a single crystal like texture corresponding to the 111, 311, 331, 422 and 511 reflections of the Si-I phase (diamond, $Fd\overline{3}m$). Under the present experimental conditions, the Si-I phase can be observed up to 13.9 GPa although a coexistence with the phases Si-II and Si-XI appears between 13.1 GPa and 13.5 GPa and will be discussed in detail later in the text.

During the data analysis, each (unsaturated) single crystal XRD peak was integrated individually and used to determine the lattice parameters for each reflection. The final value of the Si-I lattice parameter was then calculated as the average of all the measured ones.

In Table 1 the obtained results for Si-I are reported together with the corresponding pressure measured from the ruby fluorescence method^28^. Pressure measured from the compression curves of Au^31^ and W^28^ are also reported in the table to compare the reliability of the measured pressure and, to further constrain the pressure distribution inside the high pressure chamber during the experiment.

**Table 1:** Measured pressures in GPa and lattice parameters of tungsten (a_*W*_), gold (a_*Au*_) and Si-I for the four experimental runs.

Run	P_*ruby*_^28^	P*au*^31^	P_*W*_^28^	a_*Au*_ (Å)	a_*W*_ (Å)	a (Å)
ST1	0.3		0.1		3.164	5.424
	0.5		0.4		3.163	5.423
	5.4		5.1		3.148	5.343
	10.1		9.5		3.134	5.279
ST3	2.1	2.1		4.062		5.392
	6.9	6.9		4.029		5.319
	11.0	10.9		4.004		5.264
	11.2	11.1		4.003		5.261
	11.3	11.2		4.002		5.260
	11.4	11.3		4.002		5.259
	11.5	11.4		4.001		5.257
	11.6	11.5		4.000		5.256
	11.7	11.6		3.999		5.255
	11.8	11.6		3.999		5.254
	11.9	11.6		3.999		5.254
	12.0	11.9		3.998		5.252
	12.6	12.4		3.995		5.245
	12.8	12.6		3.994		5.242
	13.0	12.9		3.993		5.240
	13.2	13.0		3.992		5.239
	13.3	13.1		3.991		5.239
	13.5	13.1		3.991		5.237
ST2	0.2	0.1		4.078		5.428
	0.4	0.4		4.075		5.423
	0.6	0.6		4.074		5.420
	1.7	1.6		4.066		5.404
	2.3	2.2		4.061		5.393
	3.0	2.9		4.056		5.380
	4.1	4.0		4.048		5.364
	5.5	5.5		4.038		5.340
	7.1	7.2		4.027		5.321
	8.9	8.9		4.016		5.298
	10.1	10.1		4.009		5.279
	10.6	10.5		4.006		5.273
	11.5	11.5		4.000		5.261
ST1-2	0.0					5.431
	2.2					5.393
	11.7					5.258
	12.3					5.251
	12.5					5.247
	12.6					5.246
	12.8					5.244
	12.9					5.243
	13.2					5.240

The data are listed in the order they have been taken. Experimental uncertainty on lattice parameters is lower than 0.003 Å. Uncertainty on pressure measurement increases from 0.05 GPa at 1 GPa to 2 GPa at 150 GPa if the ruby pressure scale is assumed to be correct^47^.

**Figure 1:**
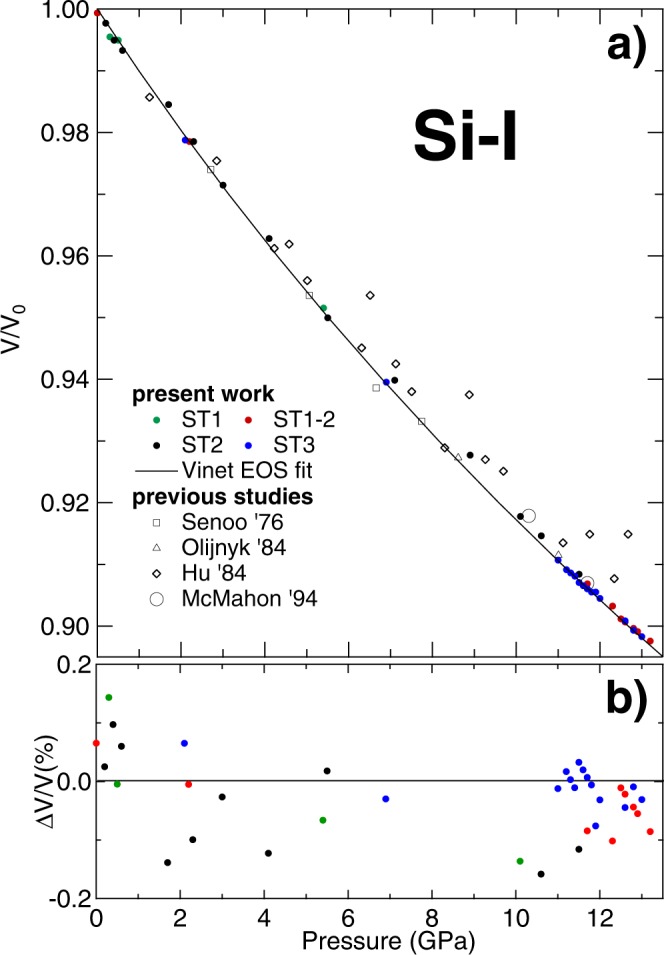
(**a**) Measured volume of Si-I as a function of pressure compared to previous experimental studies^6,10,24,36^. (**b**) Percentage error between the measured and fitted volume of the present data using a Vinet EOS with *V*_0_ = 20.031 Å^3^/atom, *K*_0_ = 97.89 GPa and ${K}_{0}^{\text{'}}$ = 4.24.

In Fig. 1 the corresponding normalized volumes per unit cell of the Si-I phase are plotted as a function of pressure together with the results obtained from previous studies. Si-I bulk modulus *K*_0_, its pressure derivative ${K^{\prime} }_{0}$ and the volume *V*_0_ at ambient pressure have been determined by a least-squares fit of the present pressure-volume data to a Rydberg-Vinet^32^ and a third-order Birch-Murnaghan (BM3) EOS^1^.

The resulting values are summarized in Table 2. The uncertainties in the obtained values correspond to the 95 % confidence interval of the fitted values. When ${K^{\prime} }_{0}$ is fixed to 4.24 as obtained in the ultrasonic measurement of *McSkimin et al*.^33^, the obtained fit values are in good agreement with the ultrasonic ones (see Table 2).

**Table 2:** EOS parameters of Si-I measured (or calculated) in different experiments.

Reference	*V*_0_ (Å^3^/atom)	*K*_0_, ${K}_{0}^{\text{'}}$ (GPa)	P range (GPa)	PTM	Pressure gauge	EOS	Method
This Study	20.021	101.5, 3.43	0.0–13.0	He	Ruby^28^, W^28^, Au^31^	Vinet	XRD in DAC
This Study	20.037	96.50, 4.24*	0.0–13.0	He	Ruby^28^, W^28^, Au^31^	Vinet	XRD in DAC
This Study	20.011	101.5, 3.45	0.0–13.0	He	Ruby^28^, W^28^, Au^31^	BM3	XRD in DAC
This Study	20.037	96.86, 4.24*	0.0–13.0	He	Ruby^28^, W^28^, Au^31^	BM3	XRD in DAC
^6^	20.012	99.90, 3.80	0.0–11.7	4:1 methanol-ethanol	Ruby^7^	BM	XRD in DAC
^33^		97.88, 4.24	0.0–0.2				ultrasonic
^24^		96.00, 3.90	0.0–9.0	1:1 ethyl-methyl-alcohol	NaCl	M	hydraulic press
^5, 36^		97.88, 4.24^+^	0.0–11.3	4:1 methanol-ethanol, Ar	Ruby^48^ or NaCl		XRD in DAC
^49^		97.83, 5.08		He	Ruby^48^		PI in DAC
^10^			0.0–8.8	4:1 methanol-ethanol	Ruby^50^		ED-XRD in DAC
^35^	19.750	94.34, 4.08					DFT with LDA
^35^	20.510	87.29, 4.06					DFT with GGA
^34^	20.070	99.10, 4.00				BM	DFT with HSE06

The volume *V*_0_, bulk modulus *K*_0_ and its pressure derivative ${K}_{0}^{\text{'}}$ are listed. Experimental methods and EOS formulation are specified. The pressure range refers to regions where only the Si-I phase is observed. *: Fixed parameter.+: According to^5^ their data are in agreement with the ultrasonic values from Mcskimin *et al*., they didn’t perform any actual fit. PTM: Pressure transmitting medium. M: Murnaghan. BM: Birch-Murnaghan. PI: Phonon Imaging.

In Table 2 the values obtained in this experiment are compared to the ones obtained from previous studies together with the observed pressure domain of pure Si-I phase. It is interesting to observe that the *K*_0_ obtained in the present study by fixing ${K}_{0}^{\text{'}}$ to the ultrasonic values of Mcskimin *at al*.^33^, results 2.7% smaller than the ones calculated by Hennig *et al*.^34^ in a DFT study using a Heyd-Scuseria-Ernzerhof screening Coulomb hybrid functional (HSE06). Whereas, the *K*_0_ values obtained with DFT calculations using a generalized gradient approximation (GGA) and a local density approximation (LDA) in Wang *et al*.^35^ are 9.5% and 2% lower than the one obtained in the present study (fixing ${K}_{0}^{\text{'}}$), respectively.

The EOS derived from the compression curve of Si-I in the present work (Fig. 1) is in good agreement with the reported curve of McMahon *et al*.^6^. We note that in that work the authors report a phase transition to Si-II at 11.0 GPa, whereas in the present study no phase transition was observed before 13.1 GPa. A good agreement is also observed with the data of Senoo *et al*.^24^ obtained with XRD experiment performed in a hydraulic press-driven cubic anvil apparatus using a 1:1 mixture of ethyl- and methyl-alcohols and, with the data obtained from an energy dispersive XRD experiment in 4:1 methanol:ethanol performed by Olijnyk *et al*.^10^.

Hu *et al*.^5,36^ also studied the compression of Si in a XRD-DAC experiment in 4:1 methanol:ethanol mixture. Comparing their result with the present study it is clear they are making an overestimation, despite their using the values derived from the ultrasonic studies of Mcskimin *et al*.^33^. Furthermore such an overestimation becomes more evident with increasing pressure with a percentage difference from the ultrasonic EOS passing from 0.27% at 3 GPa to 1.48% at 12.6 GPa. This trend is normally associated to a non-hydrostatic condition in the high pressure chamber of a DAC such as a direct compression of the sample between the diamond anvils^37^.

### Si-I, II and XI: first coexistence region

**Figure 2:**
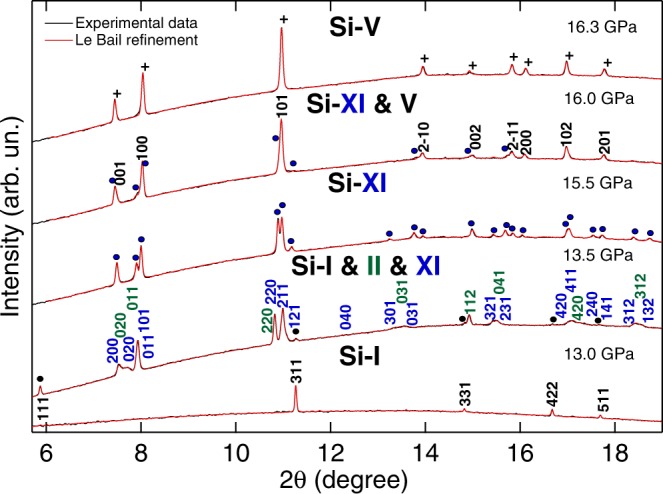
Le Bail refinements of XRD patterns collected in the ST3 run at 13.0 GPa, 13.5 GPa, 15.5 GPa, 16.0 GPa and 16.3 GPa representing the Si-I phase, coexistence between Si-I, II and XI, the Si-XI phase, coexistence between Si-XI and Si-V and the Si-V phase, respectively. All the phases have been indexed with different colours. The same colour code is used with the circle and crosses of the already indexed peaks. The 111 peak at 13.0 GPa was not included in the fitting as the single-crystal like reflection saturated the detector.

The Si-I phase (cubic diamond structure, $Fd\bar{3}m$) is stable up to a pressure of 13.1 GPa when new peaks, associated to the tetragonal *β*-tin (*I*4_1_/*amd*, Si-II) and the orthorhombic *Imma* (Si-XI) phases, develop in addition to the five reflections of the cubic diamond phase (see Fig. 2). A similar behaviour has been previously observed by McMahon *et al*.^6^, although they only discuss the possibility of a coexistence of the three phases due to the overlapping of several peaks that can belong to both the *Imma* and the *β*-tin but couldn’t be discriminated due to signal resolution problems. The coexistence of these three phases, has also been predicted by DFT simulations by Yao and Klug^38^. They found that up to a pressure of 10 GPa, the diamond structure is the most enthalpically favourable. From 10 GPa up to 13.2 GPa, the *β*-tin and the *Imma* structures are the most stable structures, both with similar enthalpies, whilst above this range the *Imma* structure becomes more favourable.

Table 3 reports the measured pressure domain of the Si-II and Si-XI phases together with the ones obtained from previous studies.

**Table 3:** Measured pressure domains for phases Si-II and XI compared to previous studies.

P domain	P′ domain	exp. type	PTM	Ref.
**Si-II**				
13.1–14.3	13.1–14.3	XRD in DAC	He	present
11.2–16.4	11.3–16.5	XRD in DAC	4:1 meth:eth	^36^
8.8–16.0	8.9–16.6	ED-XRD in DAC	4:1 meth:eth	^10^
11.7–14.4	12.0–14.8	XRD in DAC	4:1 meth:eth	^6^
11.0–13.6		DFT	N/A	^19^
**Si-XI**				
13.1–16.0	13.1–16.0	XRD in DAC	He	present
13.1–16.1	13.4–16.7	XRD in DAC	4:1 meth:eth	^6^
13.6–16.0		DFT	N/A	^19^

In the table, P refers to the reported pressure domain while P′ is the pressure domain obtained by re-interpreting each reported data using the calibration used in the present study. PTM: Pressure transmitting medium.

Under the present experimental conditions, the onset of the phase transition from Si-I to Si-II (13.1 GPa) is higher than previously observed in other static compression experiments. The corresponding pressure domain of Si-II is narrower and, actually corresponds to the observed coexistence region between Si-II and XI reported by McMahon *et al*.^6,23^. The onset of the phase transition to Si-XI is also in good agreement with the results of McMahon *et al*.^6,23^ while the observed pressure domain of Si-XI appears to be slightly narrower but in agreement within the experimental errors.

Although the *β*-tin and *Imma* phase reflections are strongly overlapping, it was possible to determine the lattice parameters for all three phases at the transition pressure via a Le Bail fit. The results obtained from the fit together with the measured pressure for both Si-II and Si-XI phases are reported in Table 4.

**Table 4:** Measured pressures in GPa and lattice parameters of gold (a_*Au*_), Si-II and XI.

P_*ruby*_^28^	P_*Au*_^31^	a_*Au*_ (Å)	phase	a(Å)	b(Å)	c(Å)
**ST3**						
13.5	13.1	3.990	II	4.648		2.560
13.6	13.2	3.990	II	4.648		2.560
13.8	13.3	3.989	II	4.647		2.559
13.9	13.5	3.988	II	4.645		2.557
14.0	13.6	3.987	II	4.644		2.558
14.1	13.7	3.987	II	4.642		2.557
13.5	13.1	3.990	XI	4.714	4.601	2.545
13.6	13.2	3.990	XI	4.714	4.603	2.543
13.8	13.3	3.989	XI	4.714	4.601	2.544
13.9	13.5	3.988	XI	4.713	4.594	2.550
14.0	13.6	3.987	XI	4.714	4.587	2.555
14.1	14.0	3.985	XI	4.716	4.586	2.554
14.3	13.9	3.986	XI	4.712	4.575	2.558
14.4	14.0	3.985	XI	4.717	4.568	2.557
14.5	14.4	3.983	XI	4.718	4.562	2.558
14.6	14.3	3.984	XI	4.723	4.554	2.558
14.7	14.6	3.982	XI	4.726	4.545	2.556
14.9	14.8	3.981	XI	4.730	4.538	2.556
15.0	14.6	3.982	XI	4.734	4.530	2.555
15.2	14.8	3.981	XI	4.737	4.520	2.555
15.3	14.9	3.980	XI	4.740	4.511	2.524
15.5	15.1	3.980	XI	4.743	4.503	2.553
15.7	15.2	3.979	XI	4.748	4.491	2.553
15.8	15.4	3.978	XI	4.748	4.479	2.551
16.0	15.5	3.977	XI	4.761	4.461	2.553
16.1	15.7	3.976	XI	4.778	4.427	2.550
**ST2**						
13.1	13.0		II	4.656		2.556
13.4	13.2		II	4.653		2.546
14.3	14.1		II	4.644		2.556
13.1	13.0		XI	4.708	4.606	2.555
13.4	13.2		XI	4.707	4.599	2.554
14.3	14.1		XI	4.700	4.591	2.561
15.3	15.2		XI	4.738	4.517	2.553
16.0	15.9		XI	4.764	4.472	2.549

Experimental uncertainty on lattice parameters is lower than 0.003 Å. Uncertainty on pressure measurement increases from 0.05 GPa at 1 GPa to 2 GPa at 150 GPa.

In Fig. 2, the reported integrated diffraction patterns and the corresponding Le Bail fits, show the structural evolution of Si from 13.0 GPa to 16.3 GPa. It is important to emphasise that at 13.5 GPa only by considering the three phases (I,II and XI) together can explain the observed reflections. Also interesting is the different behavior of the 020, 211 and 121 reflections of the Si-XI phase from 13.5 GPa to 15.5 GPa. At 13.5 GPa, when the three phases coexist, the 020 reflection of Si-XI is closer to the 200, while the 211 and 121 are overlapped, forming a single broad peak. At 15.5 GPa, however, when only the Si-XI phase is observed, the 020 reflection appears to be closer to the 101 and the 211 and 121 are well separated in two distinct peaks as previously reported^6^. Detailed XRD refinements of the coexistence region are reported from Fig. S8 to Fig. S17 of the Supplementary Materials.

**Figure 3:**
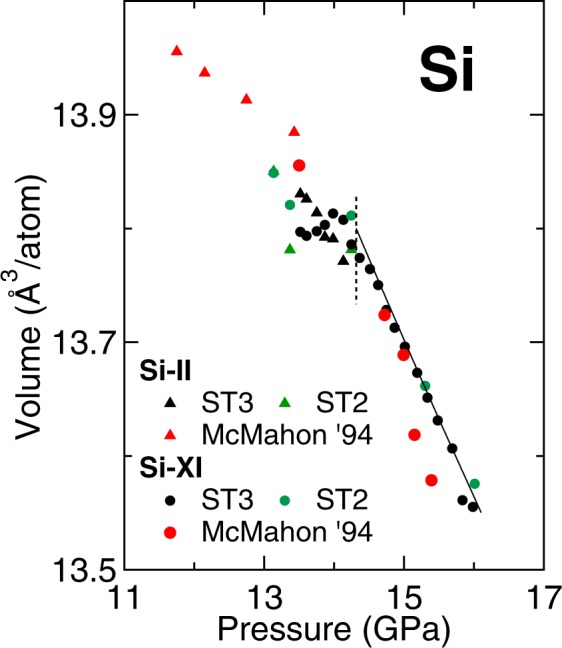
Measured volume of Si-II and Si-XI as a function of pressure (solid black and green marks) compared to previous experimental studies (red solid marks)^6^. The solid line represent a fit of the present experimental data to a Vinet EOS, whereas the dashed line separates the coexistence region from the one phase region.

In the compression curves obtained for both phases (Fig. 3) we observe a plateau corresponding to the coexistence region. In the region with only the Si-XI phase, a decreasing trend in the compression curve is observed.

### Above 16 GPa: Si-V, VI, VII and X

From 15.8 GPa, diffraction peaks for the simple hexagonal phase (*sh*; *P*6/*mmm*, Si-V) were observed in addition to those of the *Imma* phase. The two phases coexist from 15.8 to 16.1 GPa and from 16.3 GPa only the simple hexagonal phase was observed. Figure 5 shows the evolution of Si diffraction patterns at pressure above 16.3 GPa. A two phase Le Bail fit at the transition pressure of the *Imma* and simple hexagonal phases gave lattice parameters *a* = 4.748 Å, *b* = 4.479 Å, *c* = 2.551 Å (*b*/*a* = 0.943; *c*/*a* = 0.537) for *Imma*; and *a* = 2.563 Å and *c* = 2.381 Å (*c*/*a* = 0.929) for simple hexagonal. There was only a small change in volume of ~0.18% between the two phases. The phase transition from *Imma* to simple hexagonal is brought about by a further shift of the Si atoms along the [100] direction to form 6-fold symmetry chains parallel to the *Imma α*-direction/simple hexagonal *c*-direction. In the simple hexagonal structure, there is only one atom per unit cell (Wyckoff position 1a) which has a distorted 8-fold coordination environment (CN = 8; at 16.3 GPa 2 × 2.386Å, 6 × 2.553Å).

**Figure 4:**
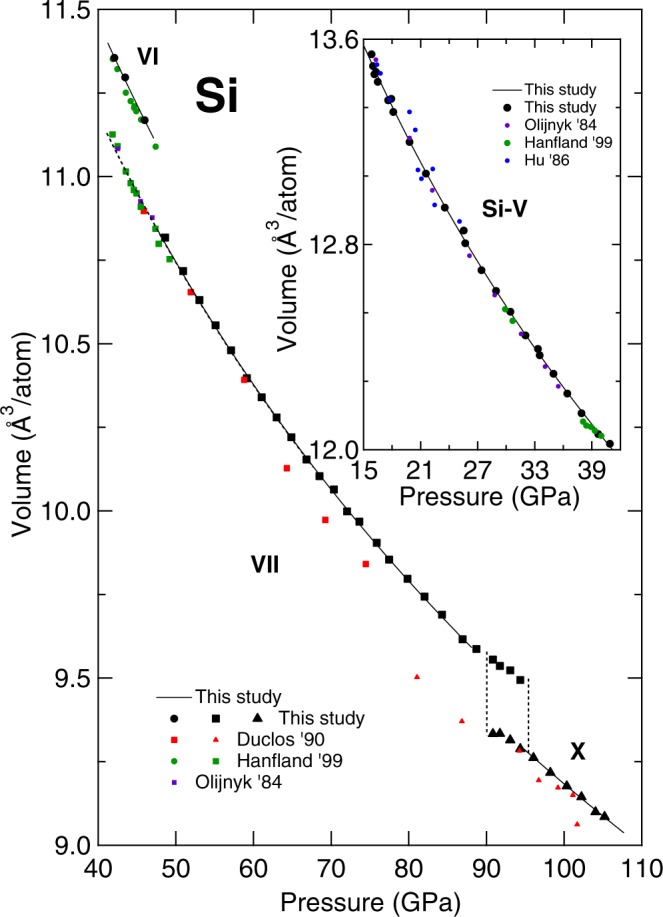
Compression curves of the Si phases observed between 40.0 GPa and 105.2 GPa (inset shows data between 15.0 GPa and 40.0 GPa) and compared to previous studies. Solid black points represent the present experimental data whereas solid black line (with the only exception of the Si-VI) are the corresponding Vinet EOS. The coexistence region between the Si-VI as Si-VII is represented by a dashed line extrapolated from the Si-VII EOS as it was not possible to measure the actual volume of Si-VII having only two peaks. The vertical dashed lines underline the coexistence region between Si-VII and Si-X phases.

**Figure 5:**
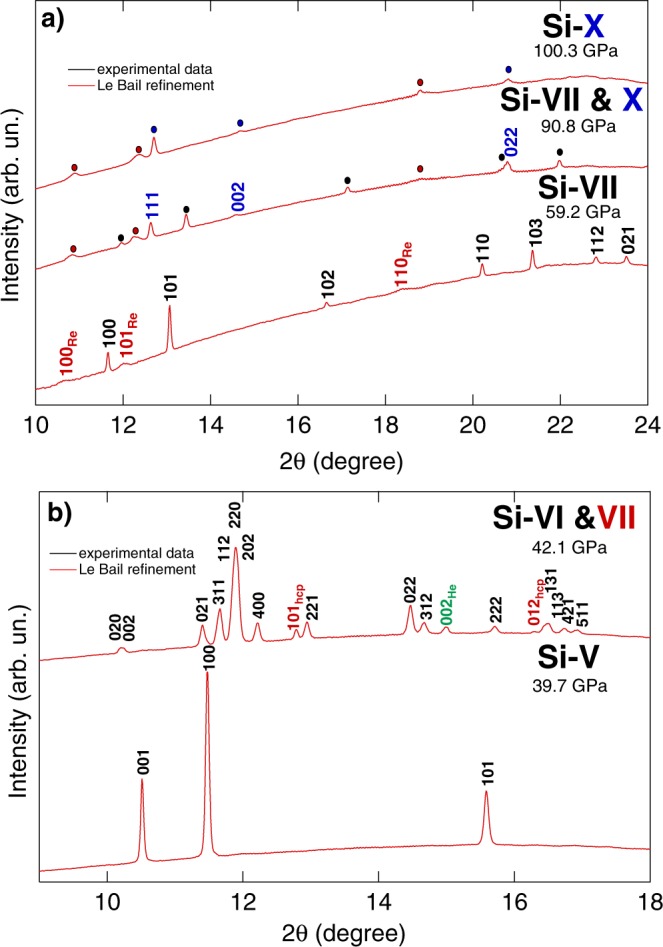
Diffraction patterns of Si collected at (**b**) 39.7 GPa, 42.1 GPa, (**a**) 59.15 GPa, 90.83 GPa and 100.3 GPa. Each pattern has been indexed and the corresponding phase(s) has(have) been specified with different colours. The same colour code is applied to the circle indicating already indexed peaks.

The simple hexagonal phase (Si-V) has been indexed up to 40.9 GPa. At 42.1 GPa, Si-V is replaced by the coexistence of Si-VI and Si-VII, the first with an orthorombic (*Cmca*) symmetry and the latter with a hexagonal (*P*6_3_/*mmc*) symmetry. This field of coexistence serve as transition to the pure Si-VII stability field that begins at 47.0 GPa (see Table 5). Si-VII is the only phase observed up to a pressure of 90.8 GPa, at which point peaks belonging to the cubic $Fm\bar{3}m$ make their first appearance together with the hexagonal structure. From 94.0 GPa, only the cubic (Si-X) phase is observed. The resulting compression curves are plotted in Fig. 4 together with previous results. Whereas, the measured lattice parameters and the corresponding pressures are reported in Tables 6 and 7. The obtained stability fields are in agreement with the literature studies^6,10,16^. However, a shift to higher transition pressure is observed in the present study (with the only exception of the study of Hanfland *et al*.^16^). Such a behaviour is attributed to the different hydrostatic condition experienced by the sample in various experiments.

**Table 5:** Measured pressure domains for phases Si-V, VI, VII and X compared to previous studies.

P domain	P′ domain	exp. type	PTM	Ref.
**Si-V**				
15.8–40.9	15.8–40.9	XRD in DAC	He	this
13.2–36.0	13.3–36.9	XRD in DAC	4:1 meth:eth	^36^
30.0*–40.0	30.6–41.1	XRD in DAC	Ar	^16^
16.0–34.0**	16.6–37.0	ED-XRD in DAC	4:1 meth:eth	^10^
15.4–16.2*	15.9–16.8*	XRD in DAC	4:1 meth:eth	^6^
36.0*–41.0	36.4*-41.7	ED-XRD in DAC	None	^17^
16.0–33.2		DFT	N/A	^19^
**Si-VI**				
42.1–46.0	42.1–46.0	XRD in DAC	He	this
38.0–47.5	39.0–49.0	XRD in DAC	Ar	^16^
39.0–43.3	39.5–44.0	ED-XRD in DAC	None	^17^
34.0**	37.0**	ED-XRD in DAC	4:1 meth:eth	^10^
33.2–40.6		DFT	N/A	^19^
**Si-VII**				
42.1–94.4	42.1–94.4	XRD in DAC	He	this
40.0–49.2*	41.1–50.8*	XRD in DAC	Ar	^16^
36.0–42.0*	36.9–43.2*	XRD in DAC	4:1 meth:eth	^36^
41.8–79.0	42.4–81.9	ED-XRD in DAC	None	^17^
40.6–80.0		DFT	N/A	^19^
**Si-X**				
90.8–105.2*	90.8–105.2*	XRD in DAC	He	this
79.0–248.0*	81.9–270.0*	ED-XRD in DAC	None	^17^
80.0–2800.0		DFT	N/A	^19^

In the table, P refers to the reported pressure domain while P′ is the pressure domain obtained by re-interpreting each reported data using the calibration used in the present study. PTM: Pressure transmitting medium.*: the reported experiment starts or ends at this pressure values. **: Higher pressure are not specified in the reported paper.

**Table 6:** The unit-cell parameters of the high-pressure phases of Si at ambient temperature.

Run	P_*ruby*_^28^	P_*Au*_^31^	P_*W*_^28^	a_*Au*_	a_*W*_	a_*V*_	c_*V*_	a_*VI*_	b_*VI*_	c_*VI*_	a_*VII*_	c_*VII*_	a_*X*_
*ST*1	17.9		17.3		3.112	2.547	2.380						
	25.5		23.3		3.096	2.510	2.357						
	33.3		32.4		3.073	2.476	2.334						
	40.9		39.8		3.056	2.449	2.315						
	43.5		42.5		3.050			7.962	4.774	4.755			
	46.0		45.0		3.045			7.921	4.758	4.742			
	48.6		47.7		3.039						2.451	4.160	
	50.9		50.1		3.034						2.443	4.146	
	53.0		52.3		3.030						2.437	4.135	
	55.1		54.4		3.026						2.431	4.125	
	57.1		56.4		3.022						2.425	4.115	
	59.2		58.6		3.018						2.419	4.105	
	61.1		60.3		3.014						2.415	4.096	
	63.0		62.2		3.011						2.410	4.089	
	64.9		64.3		3.007						2.405	4.081	
	66.8		66.0		3.004						2.400	4.072	
	68.5		67.9		3.000						2.396	4.064	
	70.4		69.9		2.997						2.391	4.064	
	72.1		71.7		2.994						2.388	4.051	
	73.6		73.1		2.991						2.385	4.047	
	75.9		75.2		2.988						2.381	4.036	
	77.5		76.9		2.985						2.376	4.031	
	79.8		79.1		2.981						2.372	4.023	
	82.0		81.2		2.978						2.367	4.015	
	84.3		83.6		2.974						2.363	4.008	
	86.9		86.4		2.969						2.357	3.997	
	88.7		87.8		2.967						2.354	3.994	
	90.8		89.8		2.964						2.352	3.990	3.342
	91.7		90.8		2.962						2.351	3.985	3.342
	93.1		92.1		2.960						2.350	3.981	3.340
	94.4		93.7		2.958						2.349	3.975	3.337
	96.1		95.4		2.955								3.334
	98.2		97.4		2.952								3.328
	100.3		99.3		2.950								3.323
	102.2		101.3		2.947								3.320
	104.0		103.4		2.944								3.314
	105.2		104.2		2.943								3.312
*ST*3	15.8	15.6		3.978		2.563	2.381						
	16.0	15.7		3.977		2.555	2.387						
	16.1	15.9		3.976		2.554	2.384						
	16.3	16.0		3.974		2.554	2.386						
*ST*2	16.5	16.8		3.972		2.550	2.385						
	17.6	17.6		3.968		2.545	2.382						
	18.1	18.2		3.965		2.542	2.380						
	19.8	19.8		3.957		2.534	2.375						
	21.6	21.3		3.9500		2.525	2.369						
	23.5	23.4		3.940		2.515	2.362						
	25.7	25.6		3.930		2.505	2.355						
	27.4	27.5		3.923		2.498	2.350						
	28.9	28.8		3.917		2.492	2.346						
	30.4	30.4		3.910		2.486	2.342						
	32.0	32.0		3.904		2.480	2.337						

All values are obtained using He as pressure transmitting medium. The pressures measured with the ruby fluorescence method and Au and W standards are all reported in GPa. The lattice parameters are reported in Å. Experimental uncertainty on lattice parameters is lower than 0.003 Å. Uncertainty on pressure measurement increases from 0.05 GPa at 1 GPa to 2 GPa at 150 GPa.

**Table 7:** The unit-cell parameters of the high-pressure phases of Si at ambient temperature.

Run	P_*ruby*_^28^	P_*Au*_^31^	P_*W*_^28^	a_*Au*_	a_*W*_	a_*V*_	c_*V*_	a_*VI*_	b_*VI*_	c_*VI*_	a_*VII*_	c_*VII*_	a_*X*_
*ST*2	33.5	33.4		3.899		2.474	2.333						
	35.0	34.8		3.894		2.469	2.329						
	36.4	36.6		3.887		2.463	2.325						
	37.9	38.0		3.882		2.458	2.321						
	39.7	39.5		3.877		2.452	2.317						
	42.1	42.0		3.868				7.987	4.781	4.758			

All values are obtained using He as pressure transmitting medium. The pressures measured with the ruby fluorescence method and Au and W standards are all reported in GPa. The lattice parameters are reported in Å. Experimental uncertainty on lattice parameters is lower than 0.003 Å. Uncertainty on pressure measurement increases from 0.05 GPa at 1 GPa to 2 GPa at 150 GPa.

The experimental compression curves for Si-V, Si-VII and Si-X were all fitted with both a Vinet and a BM3 EOS. A fit with the data obtained for the Si-VI phase was not possible as only three data points are available. During the fitting procedure, the *V*_0_ of the high pressure phases have been calculated by extrapolating the data to ambient pressure. The parameters obtained from the fit are reported in Table 8. Concerning the *P*6/*mmm* phase, the results obtained from the Vinet and the BM3 fit agrees within the error bars. The observed differences in the absolute values are explained by the trade-off between the bulk modulus and its first derivative in pressure. The lower *K*_0_ in the Vinet is compensated by a higher ${K}_{0}^{\text{'}}$. A *K*_0_ smaller than the one of Si-I is consistent with a typical cubic-to-hexagonal structural transition. A similar trend is also obtained for the compression curve of Si-VII, when both equations can be used to describe the experimental data within the error bars. As expected, the fitting parameters for the two hexagonal structures are close to each other with only a minimal reduction of the unit cell volume at higher pressure. In the higher pressure range there is an increment in the bulk modulus value caused by the stabilization of the cubic $Fm\bar{3}m$ phase.

**Table 8:** EOS parameters of Si-I, Si-V, VII and X as obtained in this experiment from a Vinet and a third-order Birch-Murnaghan (BM3) EOS.

	This study Vinet	This study BM3
**Si-I**		
*V*_0_ (Å^3^/atom)	20.021 (1)	20.011 (1)
*K*_0_ (GPa)	101.5 (3)	101.5 (2)
${K}_{0}^{\text{'}}$	3.43 (5)	3.45 (3)
**Si-XI**		
*V*_0_ (Å^3^/atom)	17.04 (6)	17.13 (6)
*K*_0_ (GPa)	45 (7)	42 (6)
${K}_{0}^{\text{'}}$	4 (2)	4 (1)
**Si-V**		
*V*_0_ (Å^3^/atom)	15.3 (1)	15.2 (1)
*K*_0_ (GPa)	95 (10)	99 (10)
${K}_{0}^{\text{'}}$	4.6 (5)	5.0 (6)
**Si-VII**		
*V*_0_ (Å^3^/atom)	14.3 (1)	14.3 (1)
*K*_0_ (GPa)	96.9 (9)	100.0 (8)
${K}_{0}^{\text{'}}$	4.01 (4)	4.40 (6)
**Si-X**		
*V*_0_ (Å^3^/atom)	13.3 (2)	13.3 (2)
*K*_0_ (GPa)	136 (5)	132 (6)
${K}_{0}^{\text{'}}$	4.2 (3)	3.8 (2)

The zero pressure volume *V*_0_, bulk modulus *K*_0_, and its pressure derivative ${K}_{0}^{\text{'}}$ are listed. The EOS formulations are specified.

## Conclusions

In the present study, the compression curve of Si was investigated from ambient pressure up to 105.2 GPa at ambient temperature using angular dispersive XRD in DAC. The experiment was performed in quasi-hydrostatic conditions using He as pressure transmitting medium. The hydrostaticity of the experiment was qualitatively determined from an analysis of the X-ray diffraction pattern at the highest pressure reached. This showed a maximum deviation from expected lattice parameters, caused by macroscopic stress, of ca. 0.08%, which is within the uncertainty of the experiment. The pressure experienced by the sample was measured via the ruby fluorescence method and from the measured atomic volume of W and Au using the calibration from Dorogokupets *et al*.^28^.

Seven different polymorphs of Si were observed in the investigated pressure range, in agreement with literature data^5–11,16,17,24,25^. The use of He as pressure transmitting medium had not lead to the formation of new Si phases in the investigated pressure range, although a general trend of each phase stability field shifting to higher pressure was observed. In particular, it is important to emphasise the observed behaviour of the well-known Si-I to Si-II phase transition^5–11^. In the present study the Si-II structure was never observed singularly, instead it was found to coexist in the entire pressure domain with the Si-XI phase.

**Figure 6:**
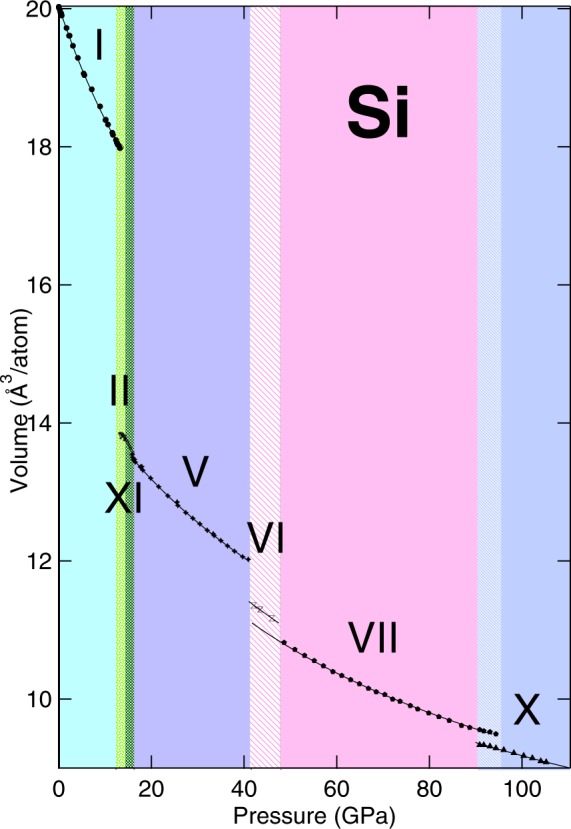
The volume-pressure relation of Si up to 105.2 GPa at room temperature using He as pressure-transmitting medium. Black symbols represents the measured experimental volumes. The continuous black line represent the fit of the experimental data of each phase to a Vinet EOS.

In Fig. 6 the measured compression curve of Si from ambient to 105.2 GPa pressure is reported. The stability fields of the different phases of Si are represented with different colors and filling pattern in the figure. The solid black lines are obtained from the fit of the measured data to Vinet EOS extrapolated down to ambient pressure. The solid line of Si-VII phase starts at 42.1 GPa, even though experimental data for this phase are not reported in the graph. The Vinet EOS curve is extrapolated into the region between 42.1 and 48.6 GPa because in this range Si-VII phase peaks were observed together with the Si-VI phase peaks, indicating their coexistence. However, as the Si-VII phase peaks were not well resolved, it was impossible to include them in a LeBail refinement.

It is interesting to notice how, due to the hydrostatic condition of the actual experiment, four different coexistence regions have been observed in the investigated pressure range. This are: the coexistence between the Si-II and the Si-XI phase in the region between 13.1 GPa and 14.3 GPa; the coexistence between the Si-XI and the Si-V phase between 15.8 GPa and 16.1 GPa; the coexistence between the Si-VI and the Si-VII phase in the region between 42.1 GPa and 46.0 GPa.

The observed coexistence between the Si-VI and the Si-VII phase is in good agreement with the data of Hanfland *et al*.^16^ although in their study, they observe a wider stability field for the Si-VI going from a coexistence with the Si-V phase at 38 GPa, to a coexistence with the Si-VII phase starting at 42.5 GPa and ending at above 47.5 GPa when only the Si-VII phase is observed.

The newly observed coexistence between the Si-VII and the Si-X phases in the region between 90.8 GPa and 94.4 GPa was probably not observed by Duclos *et al*. due to the absence of hydrostatic condition during their experiment^17^.

Finally, it is important to notice that these coexistence regions do not necessarily describe the thermodinamically stable fields since usually, solid-solid phase transitions (like those observed here) can be strongly affected by kinetic barriers^39^. Therefore, the phase boundaries experimentally determined in the present study might correspond to kinetic phase boundaries^40^. A further characterization of these coexistence region under releasing pressure will provide a better insight on the thermodynamic stability field of these phases.

## Methods

The structural evolution of Si from ambient pressure to 105.2 GPa was investigated by synchrotron XRD experiments in DAC at ambient temperature. Four DACs were used during the course of this study, each fitted with a Re gasket. Gaskets were prepared by pre-indenting Re foils (200 *μ*m initial thickness), which were then drilled by spark erosion to form the high-pressure chambers of the cells.

A grain of Si (approx 4 *μ*m each; 99.999% purity, Sigma Aldrich) was loaded into each chamber and a pressure standard was also added a few *μ*m from the Si sample. Ruby spheres were used in all four cells as pressure standards, whilst in three of them an additional W or Au X-ray standard was included since these metals have high X-ray scattering powers and well characterised EOS, attested by the consistency between static, dynamic and ultrasonic measurements^27,41^. Finally the He pressure transmitting medium was loaded into the cell. The experimental conditions adopted in each run are reported in Table 9.

**Table 9:** Condition of each experimental run. Sizes are in *μ* m; pressures are in GPa.

Run	P range	P gauge	culet size	sample size	SDD (mm)	X-rays wavelength (nm)
ST2	0.38–42.1	Ruby, Au	400	3	500.36	0.4246
ST1–2	0.32–13.3	Ruby	150 × 300	3	336.64	0.4246
ST1	0.33–105.2	Ruby, W	150 × 300	4	500.36	0.4246
ST3	2.1–16.3	Ruby, Au	500	3	470.85	0.3099

All samples were loaded in He pressure transmitting medium. SDD: Sample-to-detector distance.

Diffraction data were collected at beamline I15 (Diamond Light Source, Oxon., UK)^42^ using a monochromatic X-ray beam (*λ* = 0.4246 Å and *λ* = 0.3099 Å) and measured using a MAR345 area detector. The detector geometry was calibrated with a La *B*_6_ standard using the powder calibration routines of the DAWN software suite^43^. Measurements were made at three different sample-detector distances (500.39 mm, 424.36 mm and 324.44 mm; determined from DAWN calibration), with the distance selected depending on the angular range necessary to follow changes in the structure under investigation. Masks were applied to the raw diffraction images on a per-image basis before they were azimuthally integrated using the processing tools in the DAWN suite^44^. Diffraction data were analyzed by Le Bail fitting using the routines of the TOPAS software suite^45^, literature values for the lattice parameters of each phase were used as a starting point for these refinements.

During the experiment, pressures inside the high-pressure chambers of the DACs were measured using ruby luminescence and the unit cell volume of either W or Au for runs ST1, ST2 and ST3 and ruby luminescence only for run ST1-2. Calibration data for these three pressure standards were taken from Dorogokupets *et al*.^28^. The entire set of integrated XRD patterns is reported in Figs S1–S3 of the Supplementary Materials.

### Hydrostatic conditions

As stated in Takemura and Dewaele^31^ and Anzellini *et al*.^29^, it is desirable to achieve purely hydrostatic conditions in the sample chamber of a diamond anvil cell, which would be provided by a liquid pressure transmitting medium. However, even helium becomes solid above about 12 GPa at room temperature and above this pressure, tends to induce non-hydrostatic stress in the sample chamber. Even at lower pressure, the stress can become non-hydrostatic if the sample bridges the anvils due to excessive thinning of the gasket or due to a large initial thickness of the sample. For this reason, interferometry was used to ensure that sample size and indent depth were of the correct relative dimensions to prevent the sample bridging the diamond culets.

A qualitative analysis of the hydrostatic conditions of the sample has been performed by comparing the measured d-spacing of Si at the highest pressure reached (105.2 GPa) to the theoretical (hydrostatic) one calculated using the lattice parameter obtained by a refinement of the entire XRD pattern^26^. Table 10 shows the measured and the calculated d-spacing of Si-X at 105.2 GPa together with their percentage deviation. The deviation of less than 0.08%, is within the experimental error of the present experiment. Such an error was estimated from the deviation between the measured and calculated d-spacing of the LaB_6_ standard at ambient pressure. We can thus conclude that the non-hydrostatic stress is below the detection limit of our measurement. This is in agreement with the quantification of the macroscopic non-hydrostatic stress on metals in a helium pressure transmitting medium - i.e. 0.5 GPa at 150 GPa^46^.

**Table 10:** Measured reflections for Si-X at 105.2 GPa.

hkl	d_*m*_(Å)	d_*calc*_(Å)	$\frac{{d}_{m}-{d}_{calc}}{{d}_{calc}}$ %
111	1.91245	1.91243	0.001
002	1.65748	1.65621	0.077
022	1.17065	1.17112	0.040
311	0.99885	0.99873	0.012

*hkl* are the Miller indices of the reflection. *d*_*m*_ is the corresponding measured inter-planar distance measured by individual peak fitting. *d*_*calc*_ is the inter-planar distance calculated by a fit of the whole diffraction pattern.

A similar conclusion is obtained from the analysis of the pressure evolution of the *R*_1_-*R*_2_ splitting and the full width half maximum (FWHM) of *R*_1_ ruby fluorescence peaks (Fig. S6 of the Supplementary Materials (SM)) and the pressure evolution of the FWHM and normalized d-spacing of the W pressure gauge (Fig. S7 of SM), as explained in Takemura^26^.

## Supplementary information

Supplementary information

## Data Availability

All data generated or analysed during this study are included in this published article.
