# Supplementary material for: Quasi-hydrostatic equation of state of silicon up to 1 megabar at ambient temperature
Source: Sci Rep. 2019 Oct 29;9:15537. doi: 10.1038/s41598-019-51931-1 (PMC6820762; doi:10.1038/s41598-019-51931-1)
Supplement: Supplementary file 1 — Supplementary information [file 41598_2019_51931_MOESM1_ESM.pdf]

Supplementary Materials for

Quasi-hydrostatic equation of state of silicon up to 1 megabar at ambient temperature

Simone Anzellini[1,*], Michael T. Wharmby[1,2], Francesca Miozzi[3], Annette Kleppe[1], Dominik Daisenberger[1] and Heribert Wilhelm[1]

[1]Diamond Light Source Ltd, Diamond House, Harwell Science Campus, Didcot, Oxfordshire OX11 0DE, UK.
[2]PETRA III, Deutsches Elektronen-Synchrotron (DESY), Notekestraβe 85, 22607 Hamburg, Germany
[3]Sorbonne Université, Muséum National d'Histoire Naturelle, UMR CNRS 7590, IRD, Institut de Minéralogie, de Physique des Matériaux et de Cosmochimie, IMPMC, 75005 Paris, France.
[4]Helmotz Institut Ulm, Helmotzstraβe 11, D-89081 Ulm, Germany.

[*]simone.anzellini@diamond.ac.uk

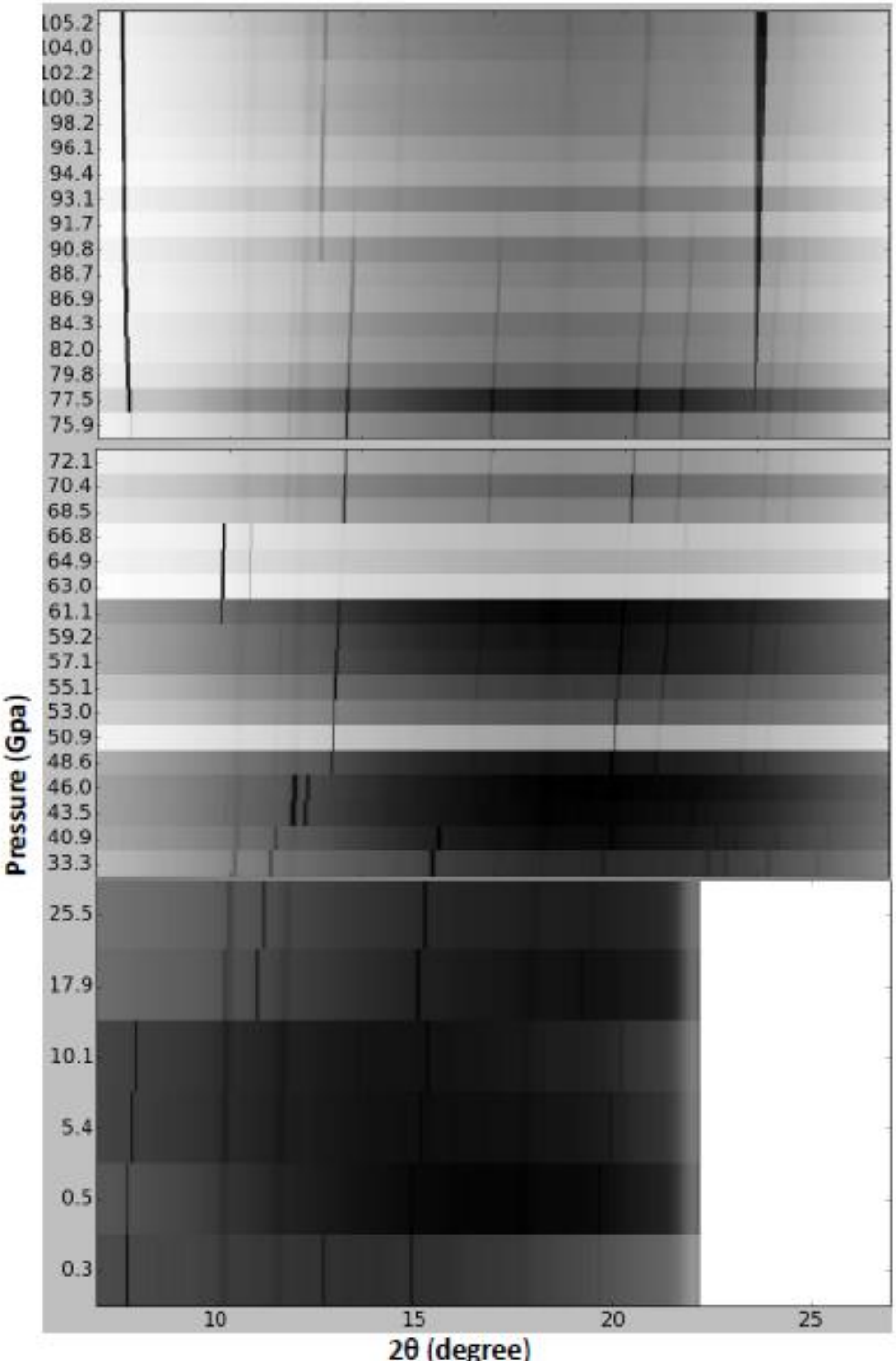

Figure S1. Evolution of all the diffraction patterns collected in run ST1 as a function of pressure

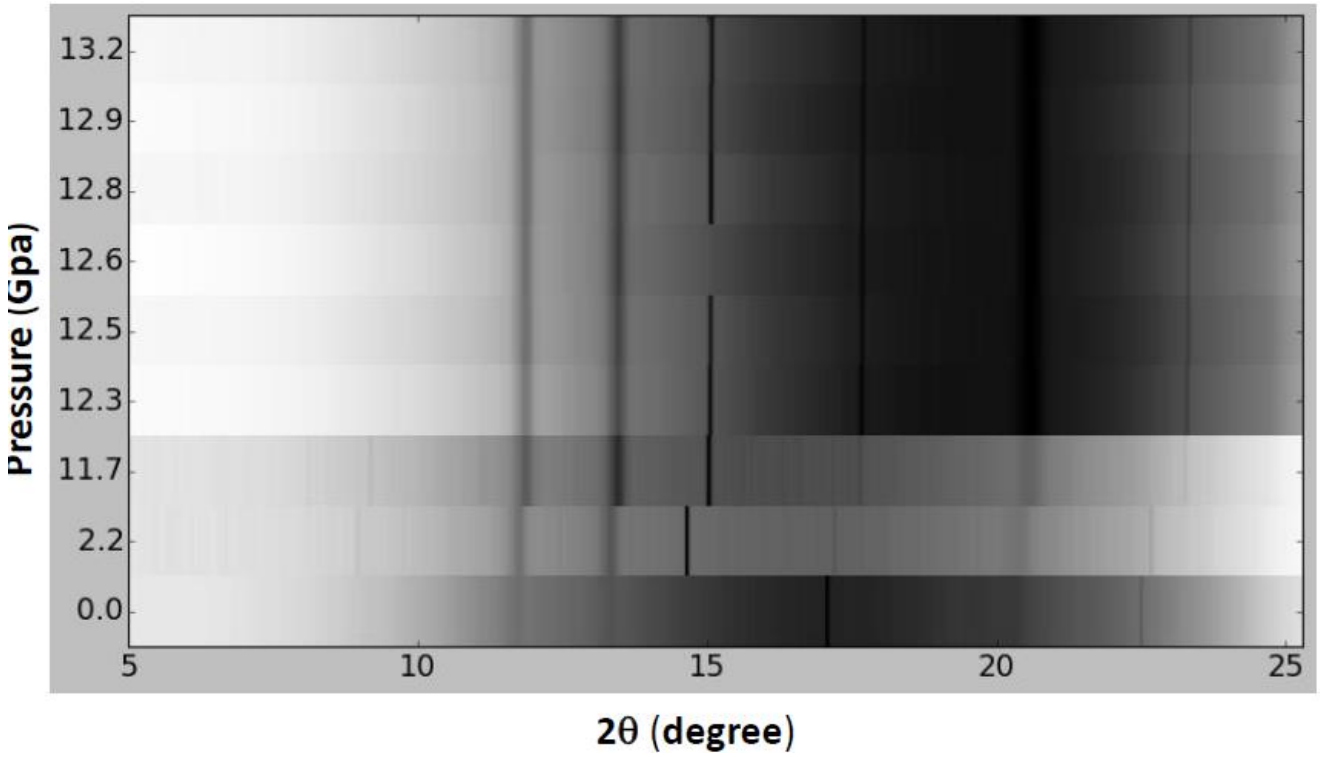

Figure S2. Evolution of all the diffraction patterns collected in run ST1-2 as a function of pressure

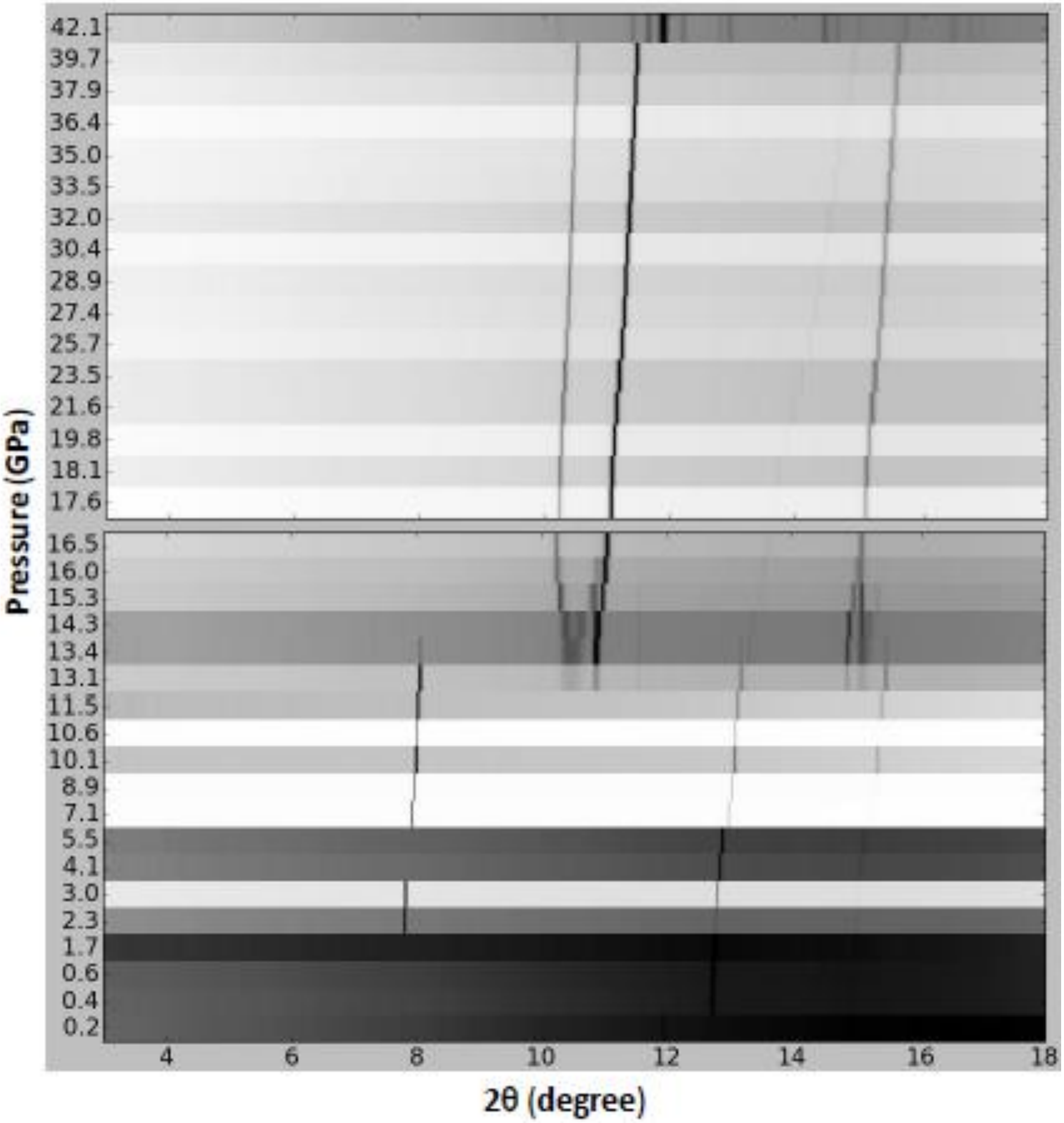

Figure S3. Evolution of all the diffraction patterns collected in run ST2 as a function of pressure

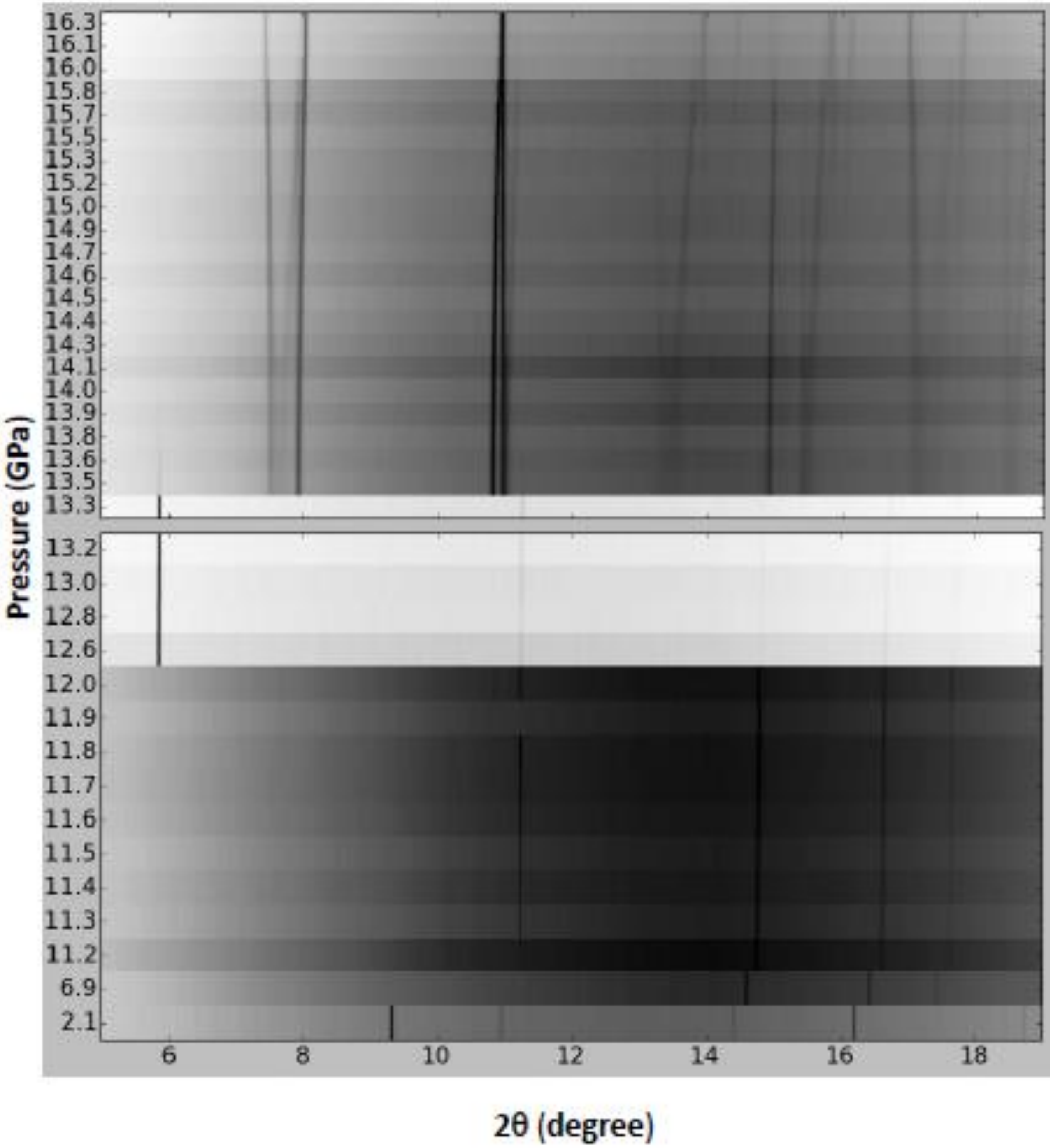

Figure S4. Evolution of all the diffraction patterns collected in run ST3 as a function of pressure

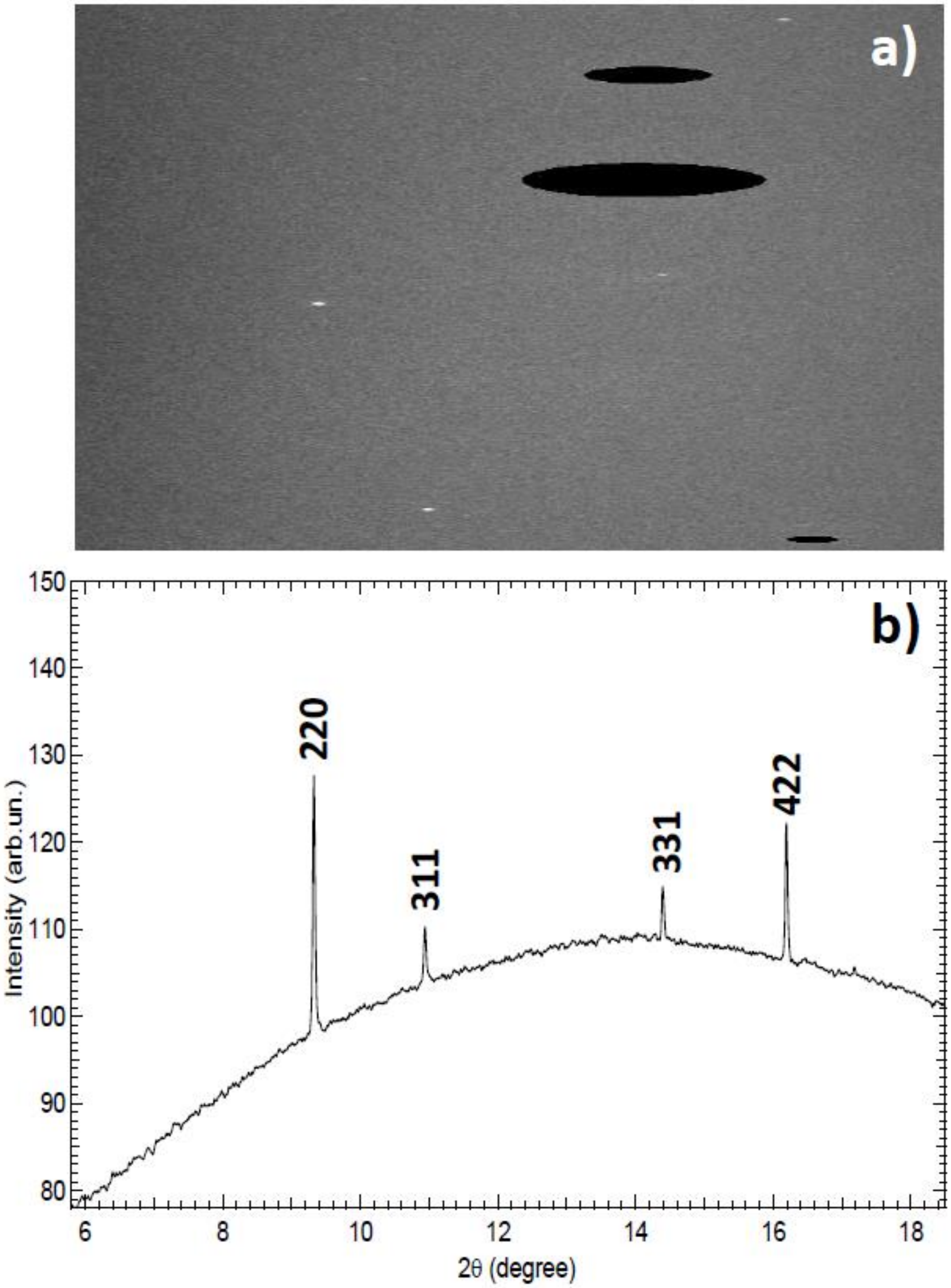

Figure S5. (a) Azimuthally unwrapped 2D diffraction image and (b) corresponding 1D integrated profile representing the texture and structure of the Si sample as obtained at the beginning of run ST3. The black ellipsoid in (a) are masks used to eliminate the saturated XRD peaks from the diamond anvils.

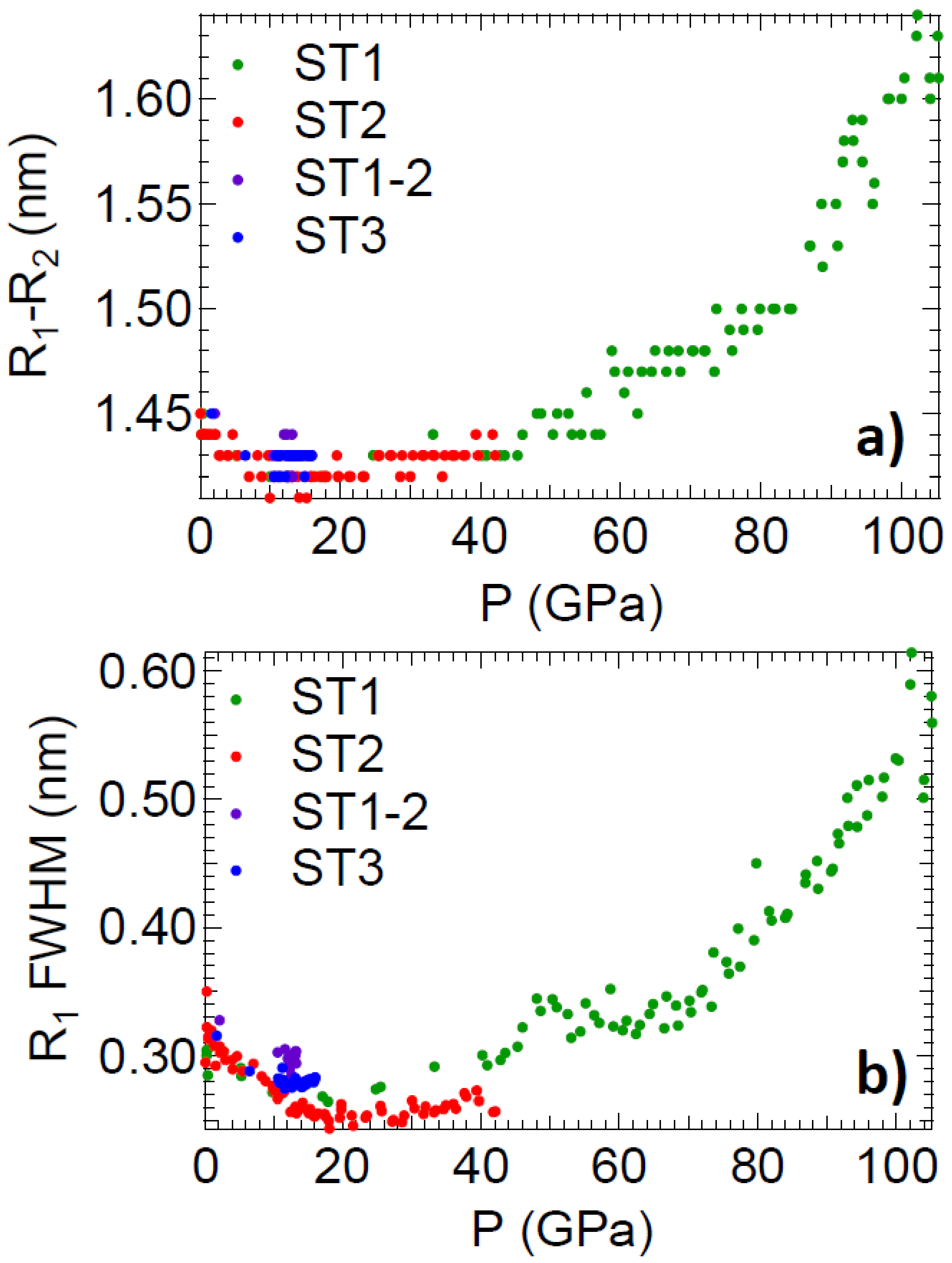

Figure S6. Pressure evolution of the (a) R_1-R_2 splitting and (b) full width half maximum of the R_1 ruby fluorescence peaks as measured in all the reported runs.

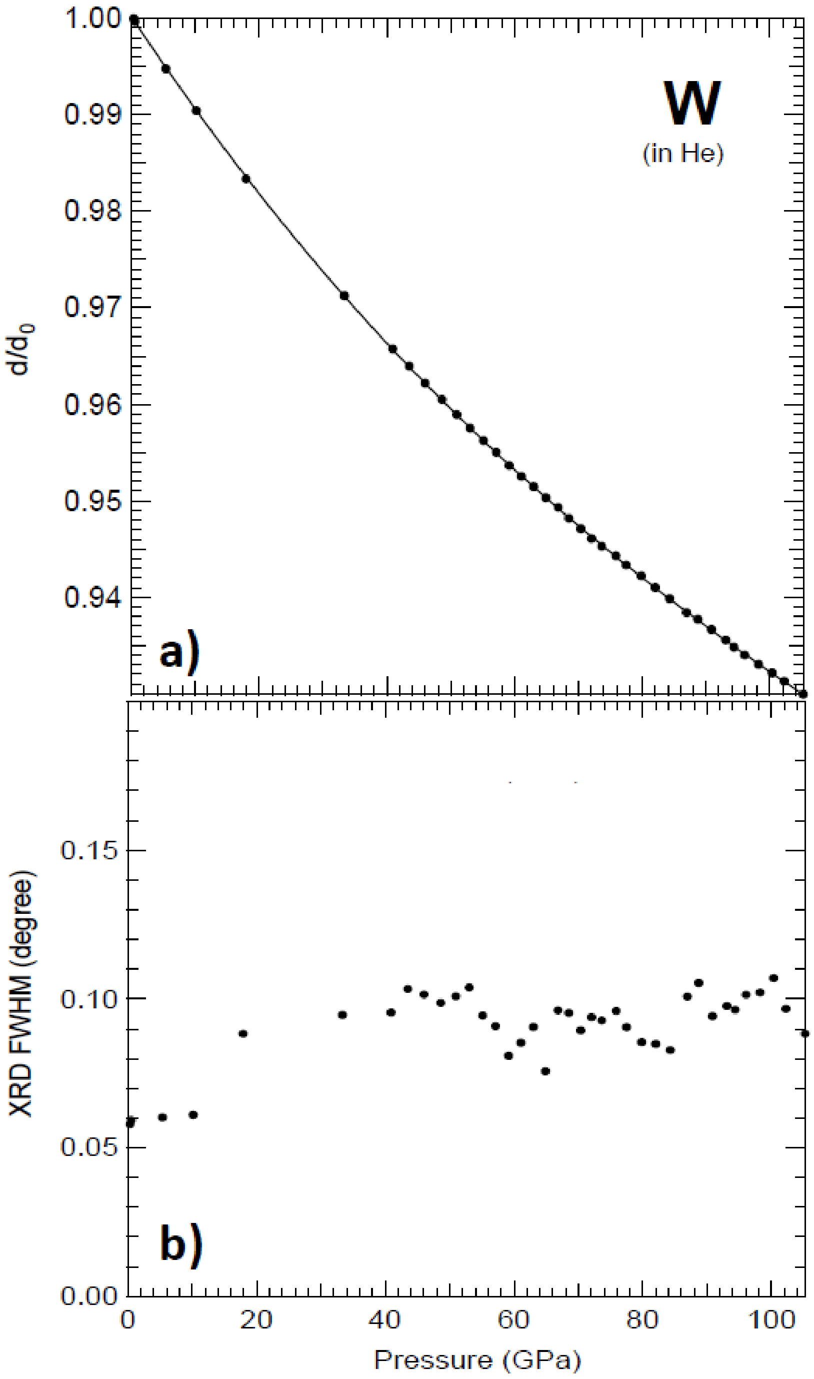

Figure S7. Pressure evolution of the (a) normalized d-spacing and (b) full width half maximum of the XRD peaks of the W pressure gauge used in run ST1. According to the method described in Takemura (2001),[26] the obtained results show that the sample was under quasi-hydrostatic condition during the entire pressure range.

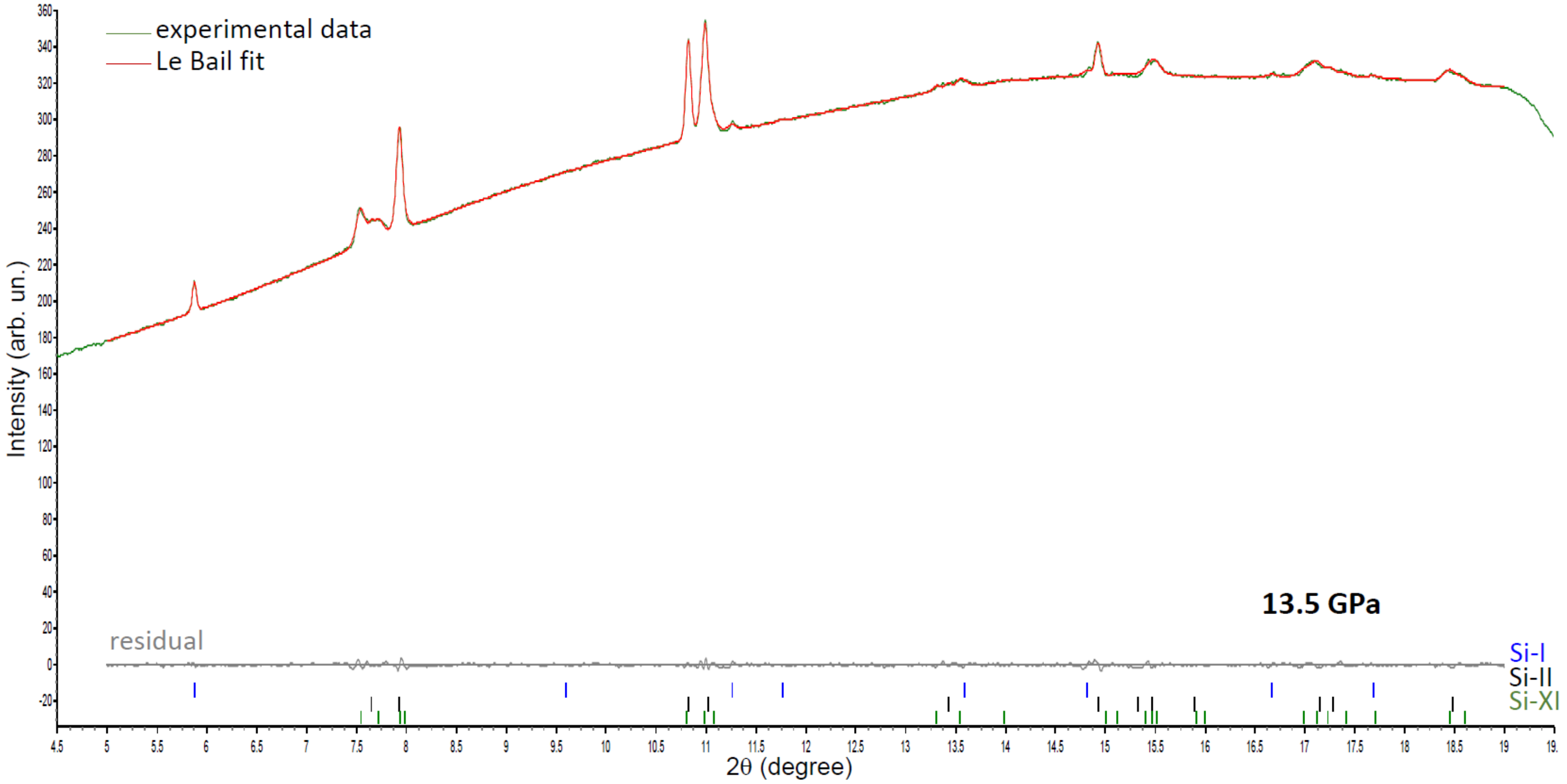

Figure S8. Refinement of the XRD pattern of Si obtained in run ST3 at 13.5 GPa. The experimental data (green line), the Le Bail fit (red line) and the corresponding residual (grey line) are represented. The resulting agreement indexes are shown in different colours at the bottom of the figure with the corresponding labels (bottom right) represented using the same colour code.

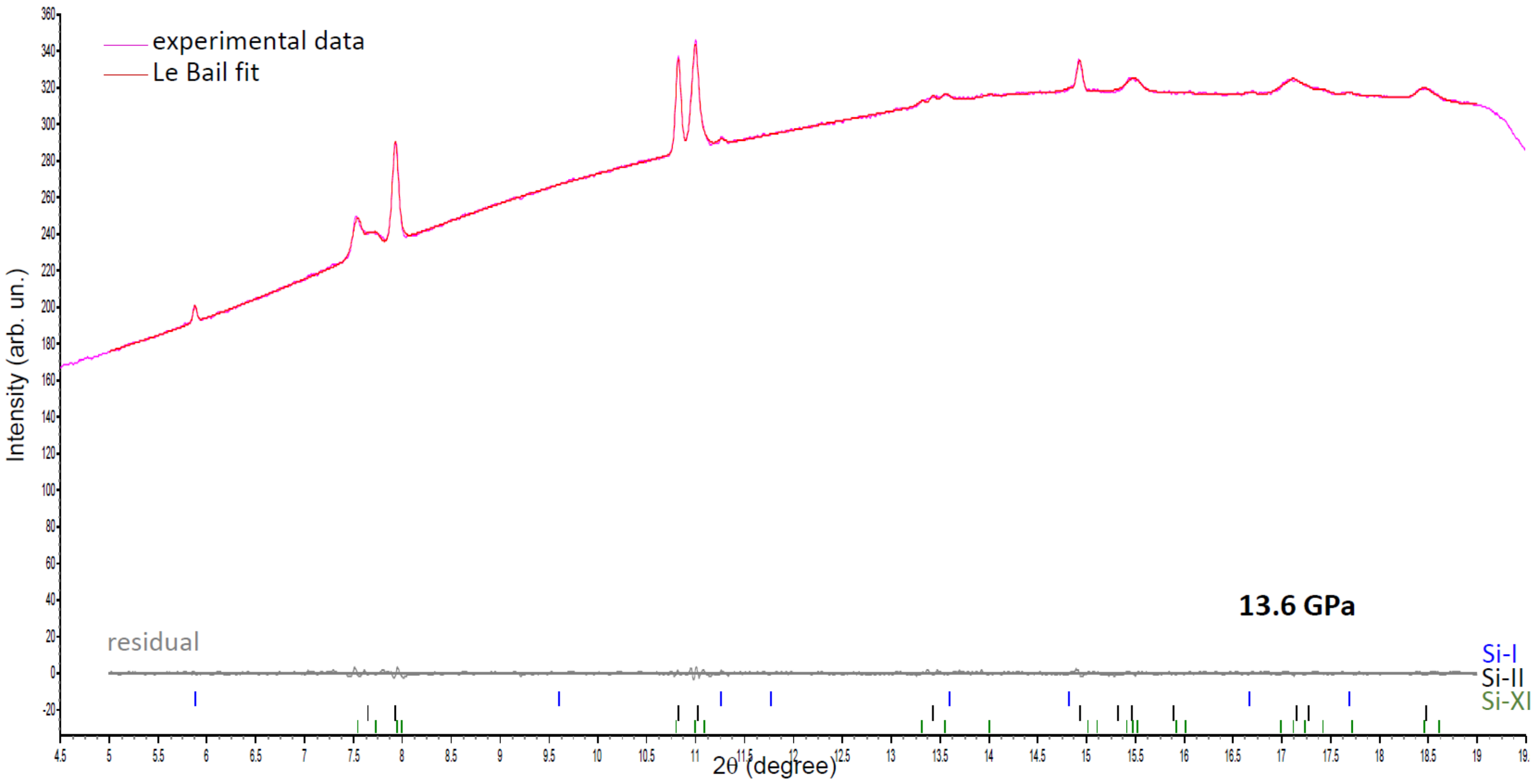

Figure S9. Refinement of the XRD pattern of Si obtained in run ST3 at 13.6 GPa. The experimental data (purple line), the Le Bail fit (red line) and the corresponding residual (grey line) are represented. The resulting agreement indexes are shown in different colours at the bottom of the figure with the corresponding labels (bottom right) represented using the same colour code.

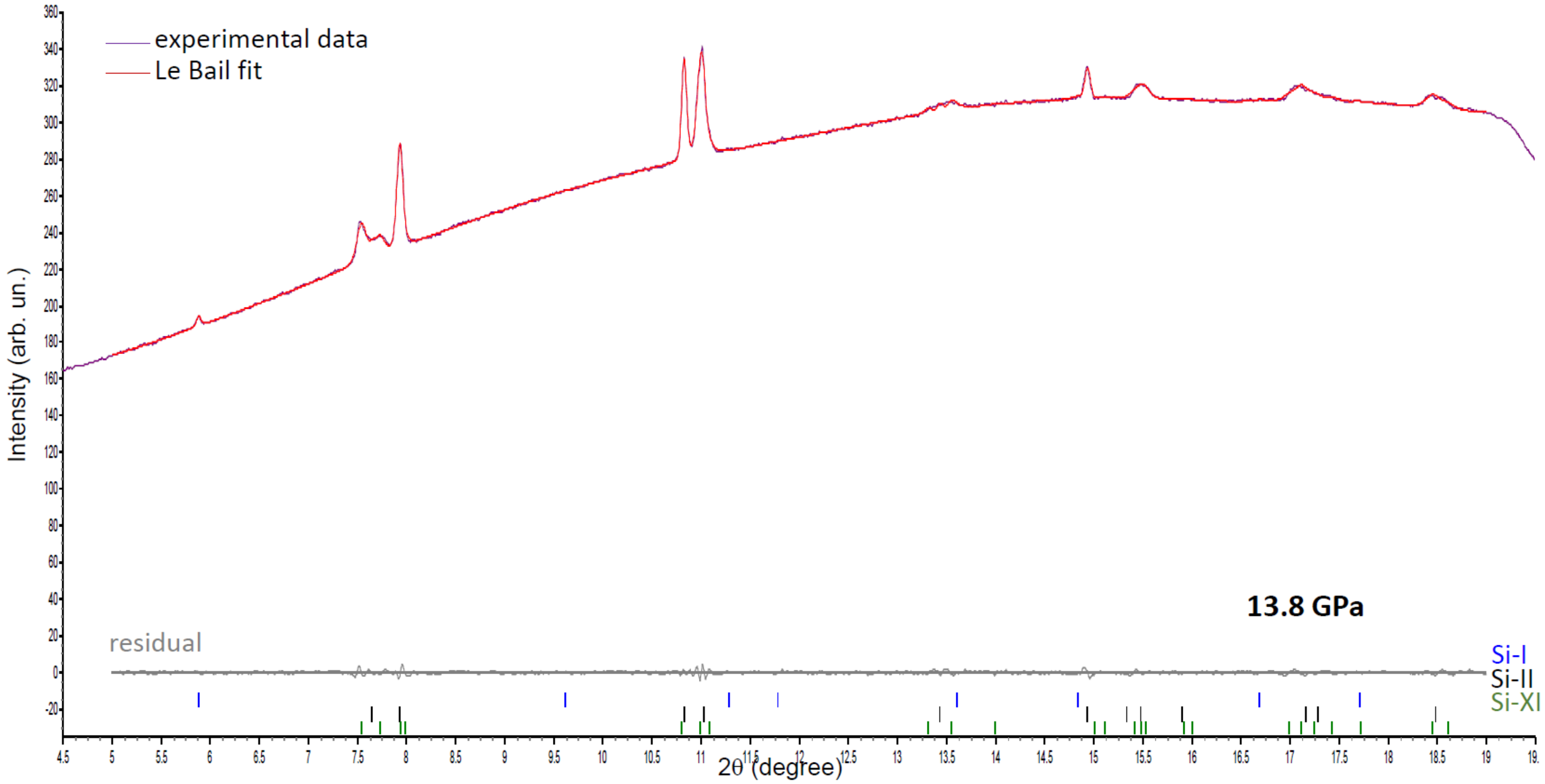

Figure S10. Refinement of the XRD pattern of Si obtained in run ST3 at 13.8 GPa. The experimental data (purple line), the Le Bail fit (red line) and the corresponding residual (grey line) are represented. The resulting agreement indexes are shown in different colours at the bottom of the figure with the corresponding labels (bottom right) represented using the same colour code.

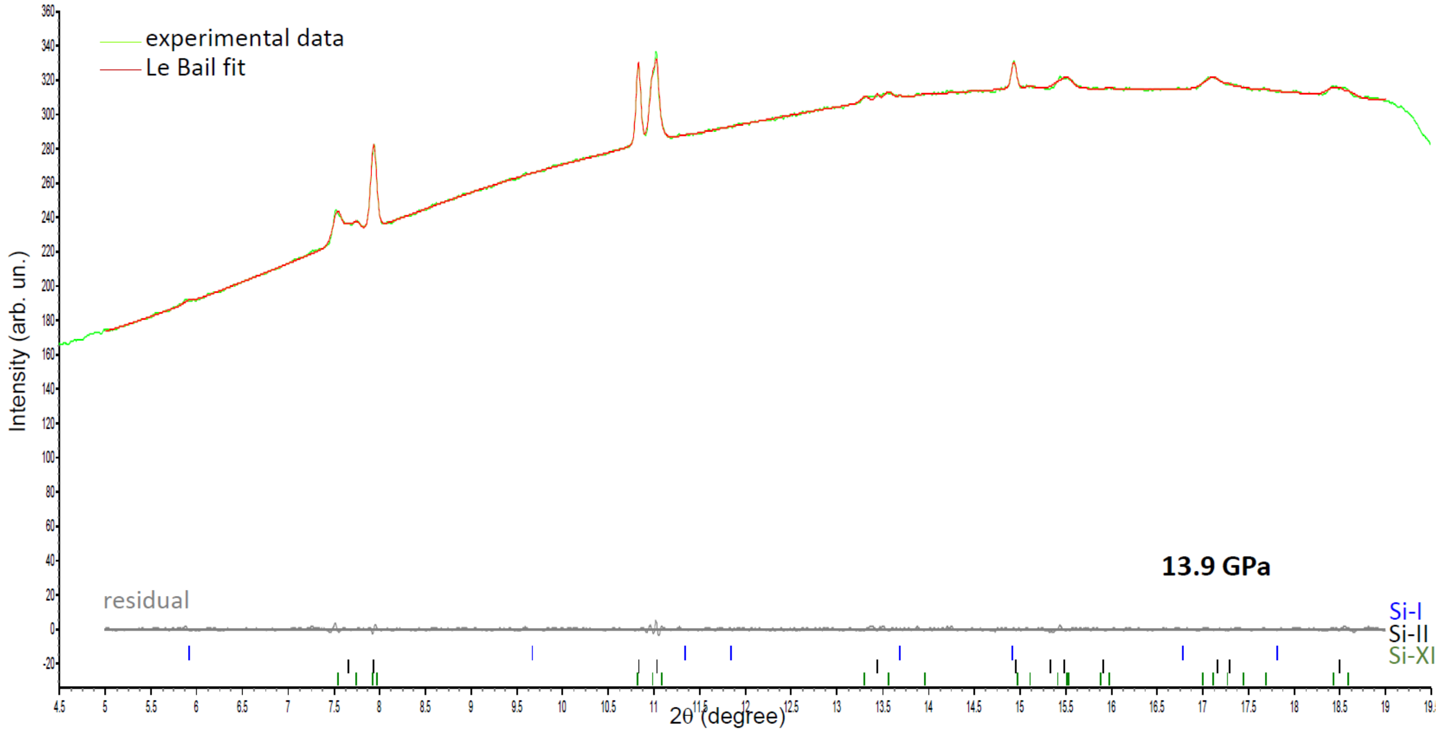

Figure S11. Refinement of the XRD pattern of Si obtained in run ST3 at 13.9 GPa. The experimental data (green line), the Le Bail fit (red line) and the corresponding residual (grey line) are represented. The resulting agreement indexes are shown in different colours at the bottom of the figure with the corresponding labels (bottom right) represented using the same colour code.

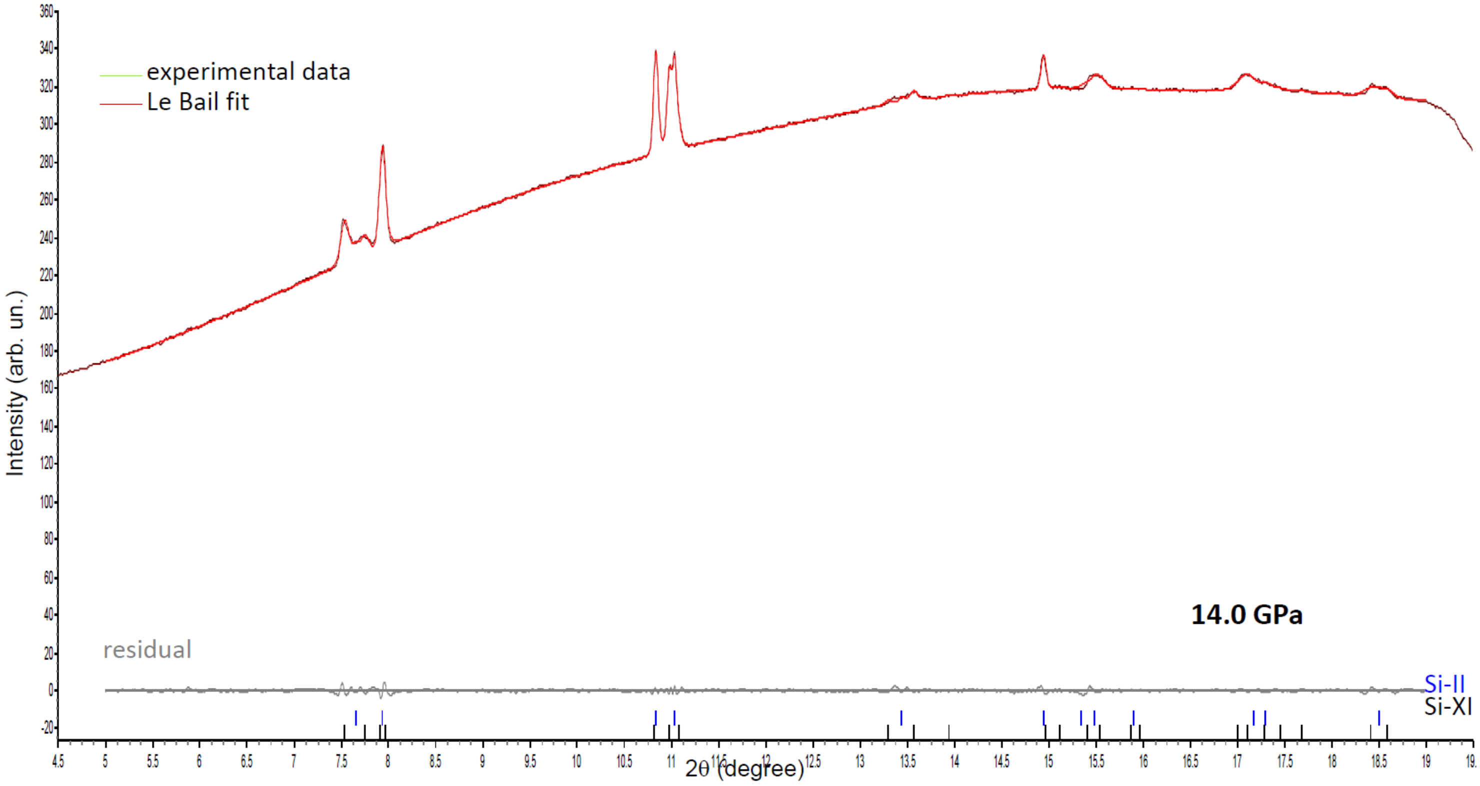

Figure S12. Refinement of the XRD pattern of Si obtained in run ST3 at 14.0 GPa. The experimental data (green line), the Le Bail fit (red line) and the corresponding residual (grey line) are represented. The resulting agreement indexes are shown in different colours at the bottom of the figure with the corresponding labels (bottom right) represented using the same colour code

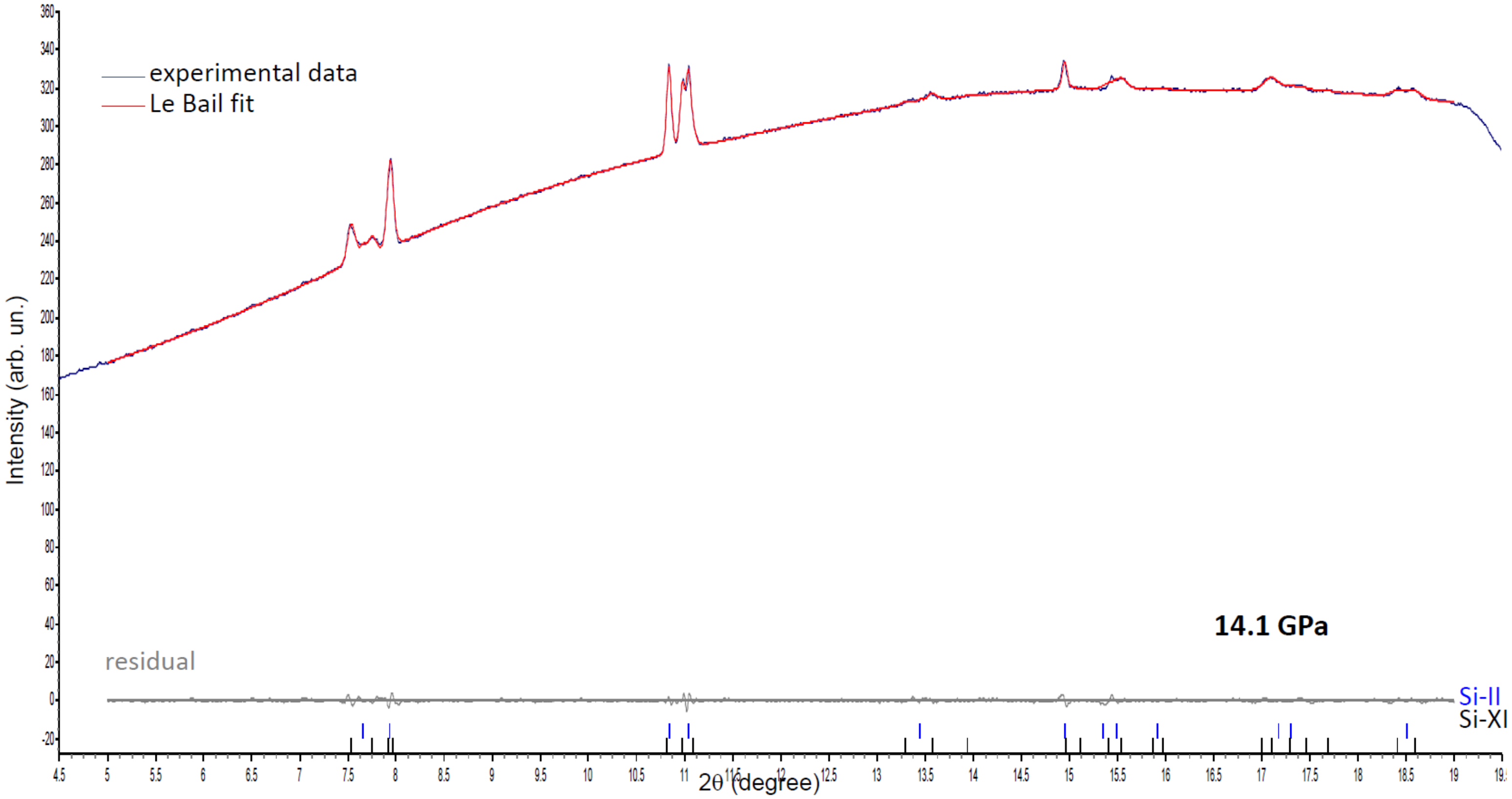

Figure S13. Refinement of the XRD pattern of Si obtained in run ST3 at 14.1 GPa. The experimental data (blue line), the Le Bail fit (red line) and the corresponding residual (grey line) are represented. The resulting agreement indexes are shown in different colours at the bottom of the figure with the corresponding labels (bottom right) represented using the same colour code

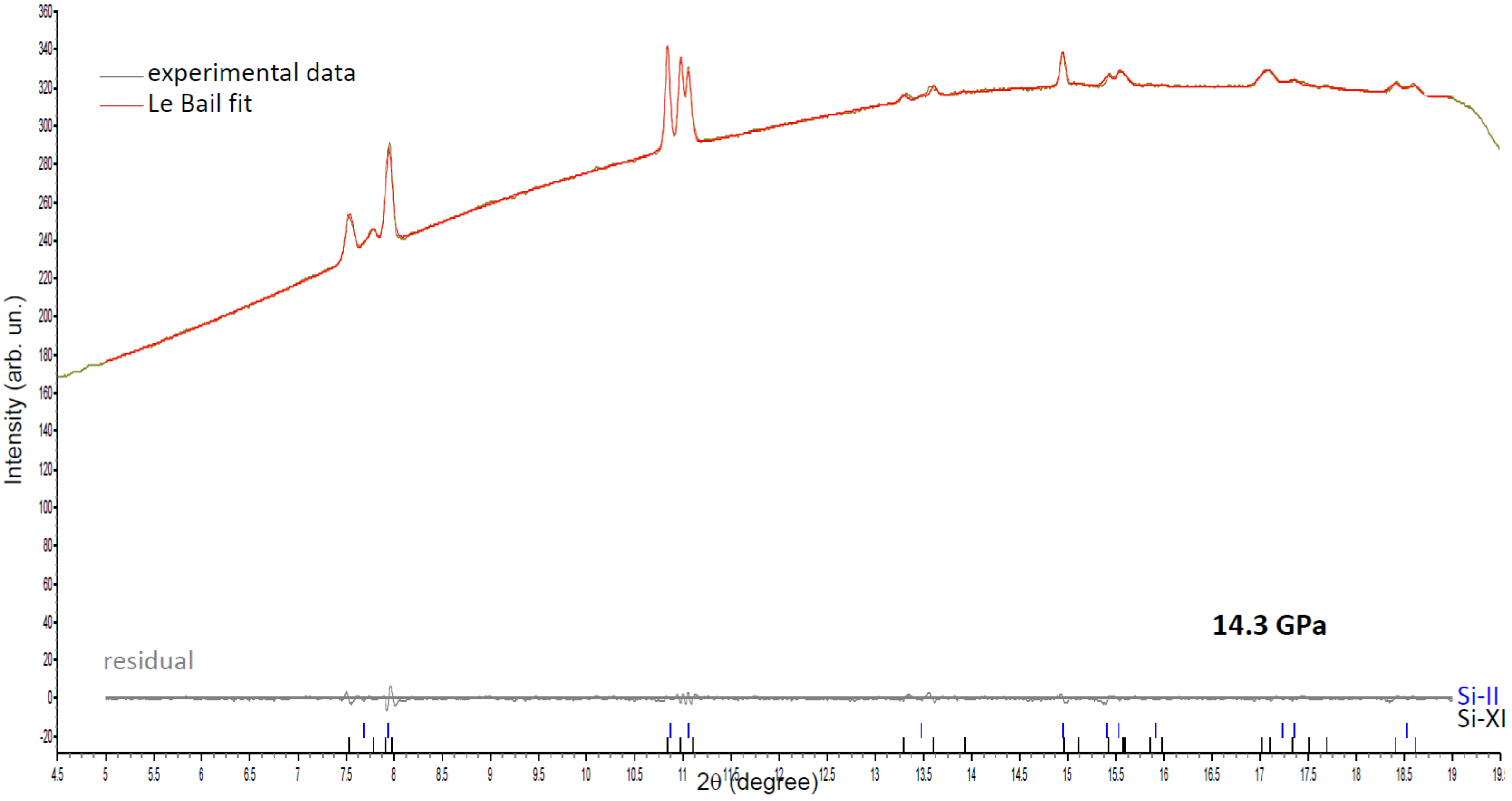

Figure S14. Refinement of the XRD pattern of Si obtained in run ST3 at 14.3 GPa. The experimental data (green line), the Le Bail fit (red line) and the corresponding residual (grey line) are represented. The resulting agreement indexes are shown in different colours at the bottom of the figure with the corresponding labels (bottom right) represented using the same colour code

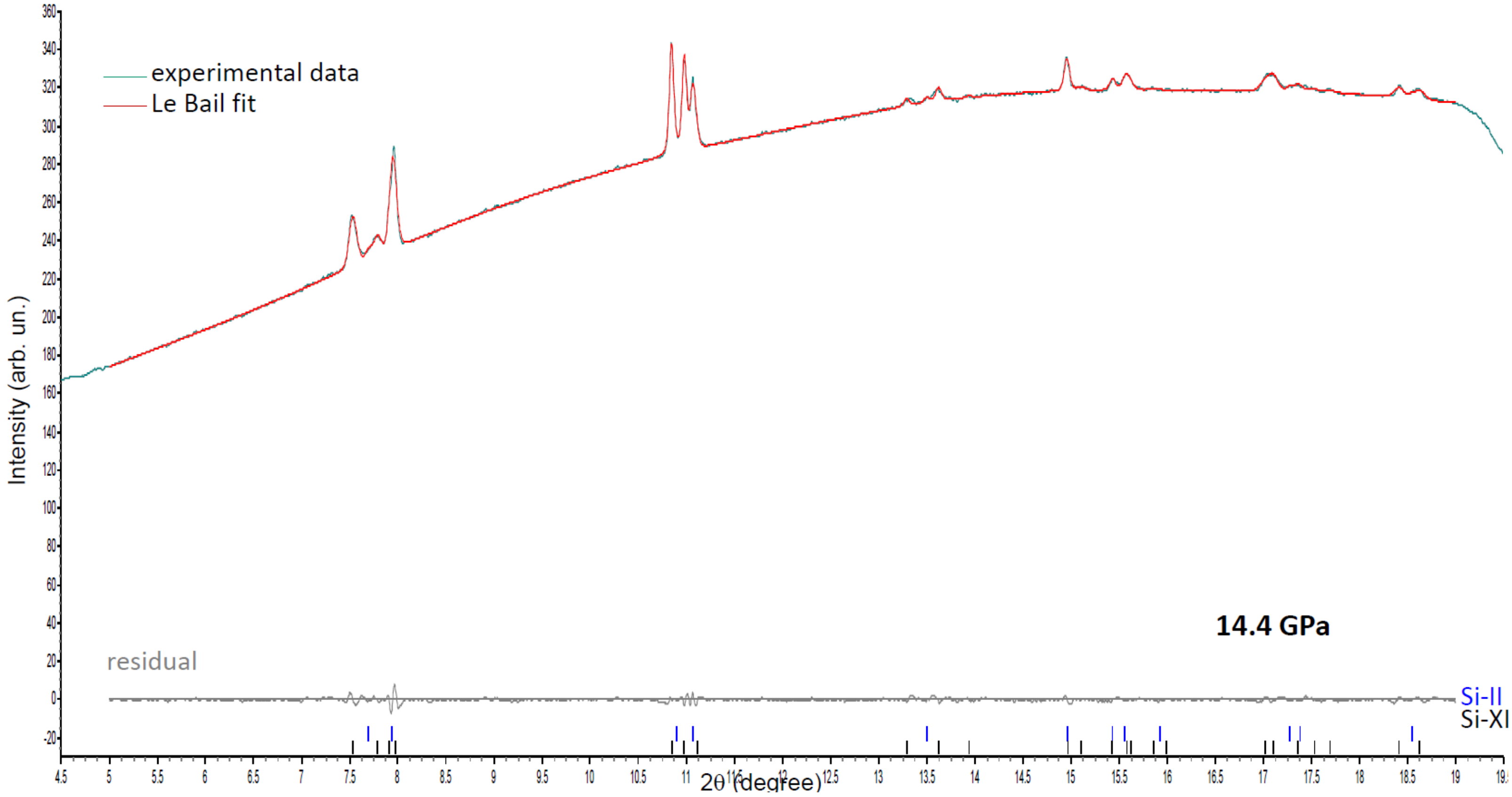

Figure S15. Refinement of the XRD pattern of Si obtained in run ST3 at 14.4 GPa. The experimental data (light blue line), the Le Bail fit (red line) and the corresponding residual (grey line) are represented. The resulting agreement indexes are shown in different colours at the bottom of the figure with the corresponding labels (bottom right) represented using the same colour code

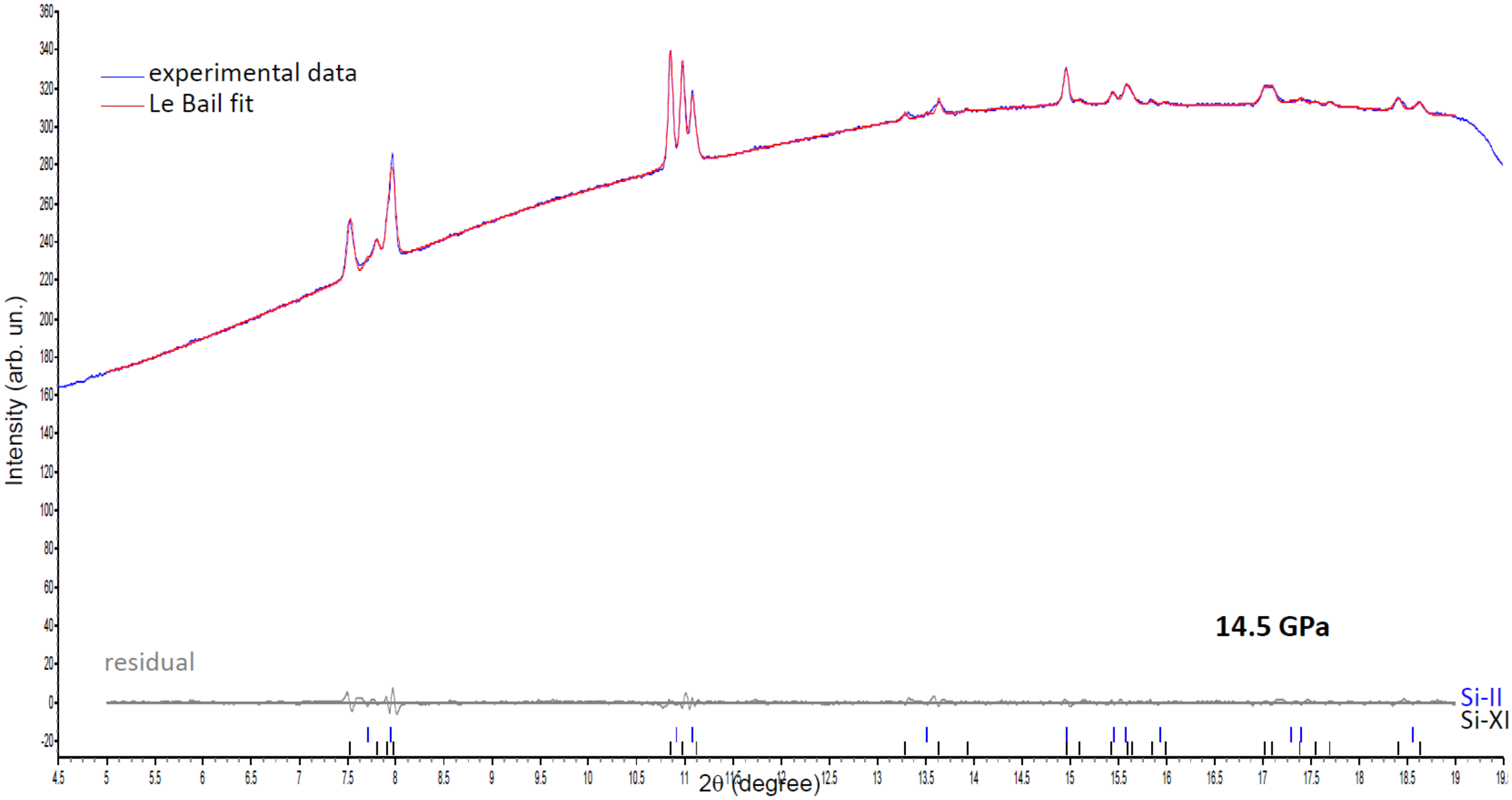

Figure S16. Refinement of the XRD pattern of Si obtained in run ST3 at 14.5 GPa. The experimental data (blue line), the Le Bail fit (red line) and the corresponding residual (grey line) are represented. The resulting agreement indexes are shown in different colours at the bottom of the figure with the corresponding labels (bottom right) represented using the same colour code

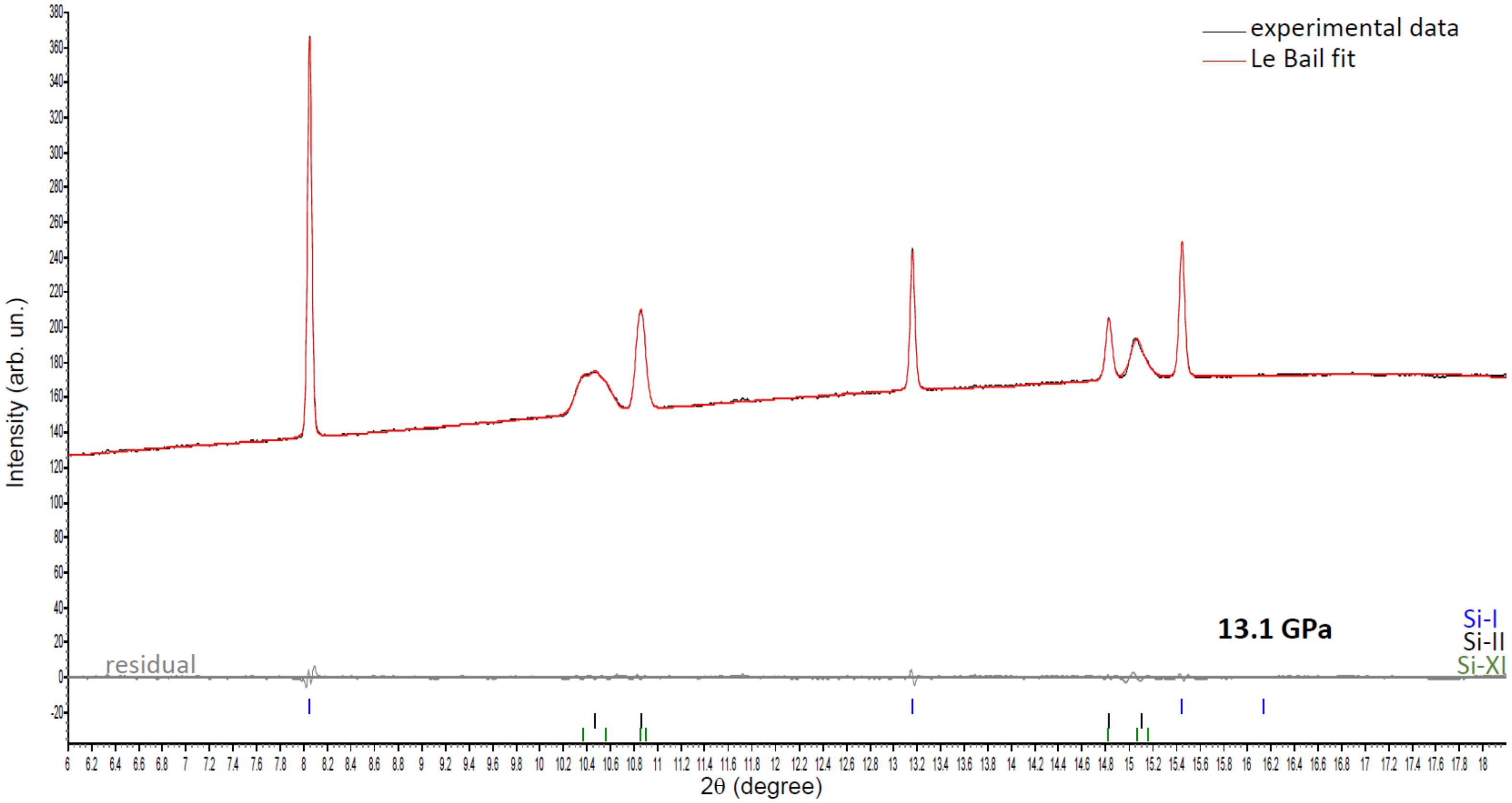

Figure S17. Refinement of the XRD pattern of Si obtained in run ST1 at 13.1 GPa. The experimental data (black line), the Le Bail fit (red line) and the corresponding residual (grey line) are represented. The resulting agreement indexes are shown in different colours at the bottom of the figure with the corresponding labels (bottom right) represented using the same colour code
